# Regulatory landscape fusion in rhabdomyosarcoma through interactions between the *PAX3* promoter and *FOXO1* regulatory elements

**DOI:** 10.1186/s13059-017-1225-z

**Published:** 2017-06-14

**Authors:** Cristina Vicente-García, Barbara Villarejo-Balcells, Ibai Irastorza-Azcárate, Silvia Naranjo, Rafael D. Acemel, Juan J. Tena, Peter W. J. Rigby, Damien P. Devos, Jose L. Gómez-Skarmeta, Jaime J. Carvajal

**Affiliations:** 1 Centro Andaluz de Biología del Desarrollo (CABD), CSIC-UPO-JA, Universidad Pablo de Olavide, Carretera de Utrera km1, 41013 Seville, Spain; 2 Division of Cancer Biology, The Institute of Cancer Research, Chester Beatty Laboratories, 237 Fulham Road, London, SW3 6JB UK

**Keywords:** TAD, CTCF, Transcriptional regulation, *FOXO1*, *PAX3*, Alveolar rhabdomyosarcoma, 4C-seq

## Abstract

**Background:**

The organisation of vertebrate genomes into topologically associating domains (TADs) is believed to facilitate the regulation of the genes located within them. A remaining question is whether TAD organisation is achieved through the interactions of the regulatory elements within them or if these interactions are favoured by the pre-existence of TADs. If the latter is true, the fusion of two independent TADs should result in the rewiring of the transcriptional landscape and the generation of ectopic contacts.

**Results:**

We show that interactions within the *PAX3* and *FOXO1* domains are restricted to their respective TADs in normal conditions, while in a patient-derived alveolar rhabdomyosarcoma cell line, harbouring the diagnostic t(2;13)(q35;q14) translocation that brings together the *PAX3* and *FOXO1* genes, the *PAX3* promoter interacts ectopically with *FOXO1* sequences. Using a combination of 4C-seq datasets, we have modelled the three-dimensional organisation of the fused landscape in alveolar rhabdomyosarcoma.

**Conclusions:**

The chromosomal translocation that leads to alveolar rhabdomyosarcoma development generates a novel TAD that is likely to favour ectopic *PAX3:FOXO1* oncogene activation in non-*PAX3* territories. Rhabdomyosarcomas may therefore arise from cells which do not normally express *PAX3*. The borders of this novel TAD correspond to the original 5'- and 3'- borders of the *PAX3* and *FOXO1* TADs, respectively, suggesting that TAD organisation precedes the formation of regulatory long-range interactions. Our results demonstrate that, upon translocation, novel regulatory landscapes are formed allowing new intra-TAD interactions between the original loci involved.

**Electronic supplementary material:**

The online version of this article (doi:10.1186/s13059-017-1225-z) contains supplementary material, which is available to authorized users.

## Background

The advent of chromatin conformation capture technologies (3C and its variants Hi-C, 5C-seq and 4C-seq; reviewed in [1]) has been essential in the identification of megabase-scale chromosomal organisation domains [2–4], which have been termed topologically associating domains (TADs). These are large genome intervals defined by an increased number of long-range chromatin interactions between the loci contained in the same chromosomal domain and a decreased number of interactions with loci in neighbouring domains [5]. Increasing experimental evidence suggests that TADs constitute not only structural but also functional units of the genome. TADs structurally restrain epigenetic domains [2–4], domains that can change coordinately in response to external cues [6]. Furthermore, the genome has been divided into compartments with active or inactive status [7], and during differentiation, regions subject to repositioning from one of these compartments to the other correspond to single or several, consecutive TADs [8, 9]. Therefore, the genes contained within a TAD, as a group, are more or less prone to transcription depending on the epigenetic state of the domain or the nuclear compartment in which they are positioned. In fact, genes within TADs do show gene expression correlation [3, 6], revealing an underlying mechanism of intra-TAD gene regulation, which does not necessarily imply that genes included within a TAD are under the control of the same tissue-specific enhancers.

From an evolutionary point of view, it has been shown that ancestral recombinations leading to loss of synteny occur at TAD borders [10], maintaining their structures and indicating that TADs are under positive selective forces, most likely because the disruption of a TAD has deleterious effects on the regulation of the genes within it. It is still not clear if TADs originate from interactions between enhancers and promoters within the domain or if it is this compartmentalisation that permits and restricts enhancer-promoter contacts [11–13].

The molecular nature of TAD borders is still unclear, although it has been shown that they are enriched in binding sites for the CTCF protein [2, 3], which has been implicated in three-dimensional (3D) chromatin organisation and enhancer-blocking activities [14]. The directionality of the CTCF binding sites seems to be predictive of their loop-forming activity as deletion or inversion of these sites results in the generation of inappropriate enhancer-promoter contacts [15, 16].

A remaining question is how sequence interactions are restricted to individual domains. The borders between adjacent TADs seem to restrict cross-border interactions and thus deletion of these regions results in the mis-regulation of the genes associated with them. Genome manipulations of the border separating the *Tfap2c* and *Bmp7* loci in the mouse show ‘contamination’ of the transcriptional landscapes of both genes upon inversion [17], while human disorders such as polydactyly, brachydactyly and F-syndrome have been shown to be related to the deletion, inversion or duplication of borders separating the different TADs containing the *WNT6-IHH/EPHA4/PAX3* loci [18], which leads to otherwise prohibited promoter contacts with enhancer elements located outside their cognate TAD, causing mis-expression of the genes involved. Analyses of various duplications in the proximity of the *SOX9* locus have shown several outcomes depending on the exact nature of the duplication: intra-TAD duplications do not alter overall TAD organisation but may result in increased numbers of intra-TAD contacts and could give rise to a phenotype; and inter-TAD duplications that cross TAD borders generate novel TADs without altering flanking gene expression. In this second case, a phenotype could arise if the novel regulatory landscape created by the duplication includes a coding gene, as it could result in its dysregulation [19].

Thus, the implication is that removal of a border element results in the fusion of adjacent TADs, while the inversion/duplication of a border could allow new regulatory interactions to be formed resulting in inappropriate expression of genes around the inversion/duplication. Importantly, sequences adjacent to the manipulated borders are also rearranged during the process and thus a possible contribution to the observed phenotypes cannot be discarded. Other human chromosomal rearrangements have been shown to result in the dysregulation of gene expression by regulatory elements located in the proximity of the breakpoints (e.g. [20–26]).

Recurrent chromosomal translocations are formed by end-joining of two double-strand chromosomal breaks, which occasionally occur within the introns of individual genes resulting in the generation of a novel chimaeric fusion protein harbouring functional domains from the two proteins and thus new functional properties. In cancer, the formation of novel chimaeric transcription factors, in which the DNA binding domain is encoded by one gene and the transactivation domain is encoded by the other, is common. The *PAX3:FOXO1* fusion gene, arising from the t(2;13)(q35;q14) translocation [27] in the paediatric soft tissue tumour alveolar rhabdomyosarcoma (ARMS), encodes a transcription factor that contains the *PAX3* (paired box 3) DNA-binding domain and the *FOXO1* (forkhead box O1) transactivation domain. This fusion transcription factor dysregulates *PAX3* target genes resulting in gene expression changes that modify pathways involved in proliferation and/or survival, contributing to tumour initiation. Translocations involving *PAX3* (or the closely related *PAX7*) and *FOXO1* are only found in rhabdomyosarcomas. This permits the formulation of two hypotheses: (1) that translocations can occur in multiple cell types but only those expressing the regulatory factors required for the expression of the oncogene give rise to rhabdomyosarcomas; or (2) that the translocations occur in a restricted or unique cell type, usually by means of co-transcription of the two loci involved in the translocation [28, 29]. Even if this second hypothesis turns out to be correct, it is still possible that only those cells that express the correct combination of transcription factors would give rise to tumour cells as the fusion gene will be under the transcriptional control of specific regulatory elements; oncogene activation in a non-*PAX3*-expressing cell type may therefore be essential for the development of the disease. It is thus clear that unravelling the transcriptional regulatory mechanisms of *PAX3*, *FOXO1* and the oncogenic *PAX3:FOXO1* gene should help to identify the elusive cell type of origin for these sarcomas.

Crucially, we show that the t(2;13)(q35;q14) translocation in ARMS not only generates a fusion gene but also a novel fused regulatory landscape that likely controls the expression of the novel gene. The translocation results in the formation of a novel TAD structure that retains the 5' and 3' borders of the wild-type *PAX3* and *FOXO1* TADs, respectively. Importantly, interactions between the *PAX3* promoter and the *FOXO1* region are similar to those established by the *FOXO1* promoter in its own locus, despite these regulatory regions being in a completely new regulatory landscape. As these interactions are novel, if the establishment of regulatory interactions were to precede TAD formation, we would expect a change in TAD boundaries. Instead, we observe that in the ARMS translocation analysed, the *PAX3* promoter does not interact with sequences downstream of the original *FOXO1* TAD border.

## Results

### Loss of synteny analyses place the 5' boundary of the *FOXO1/FoxO1* locus in close proximity to its promoter

**Fig. 1:**
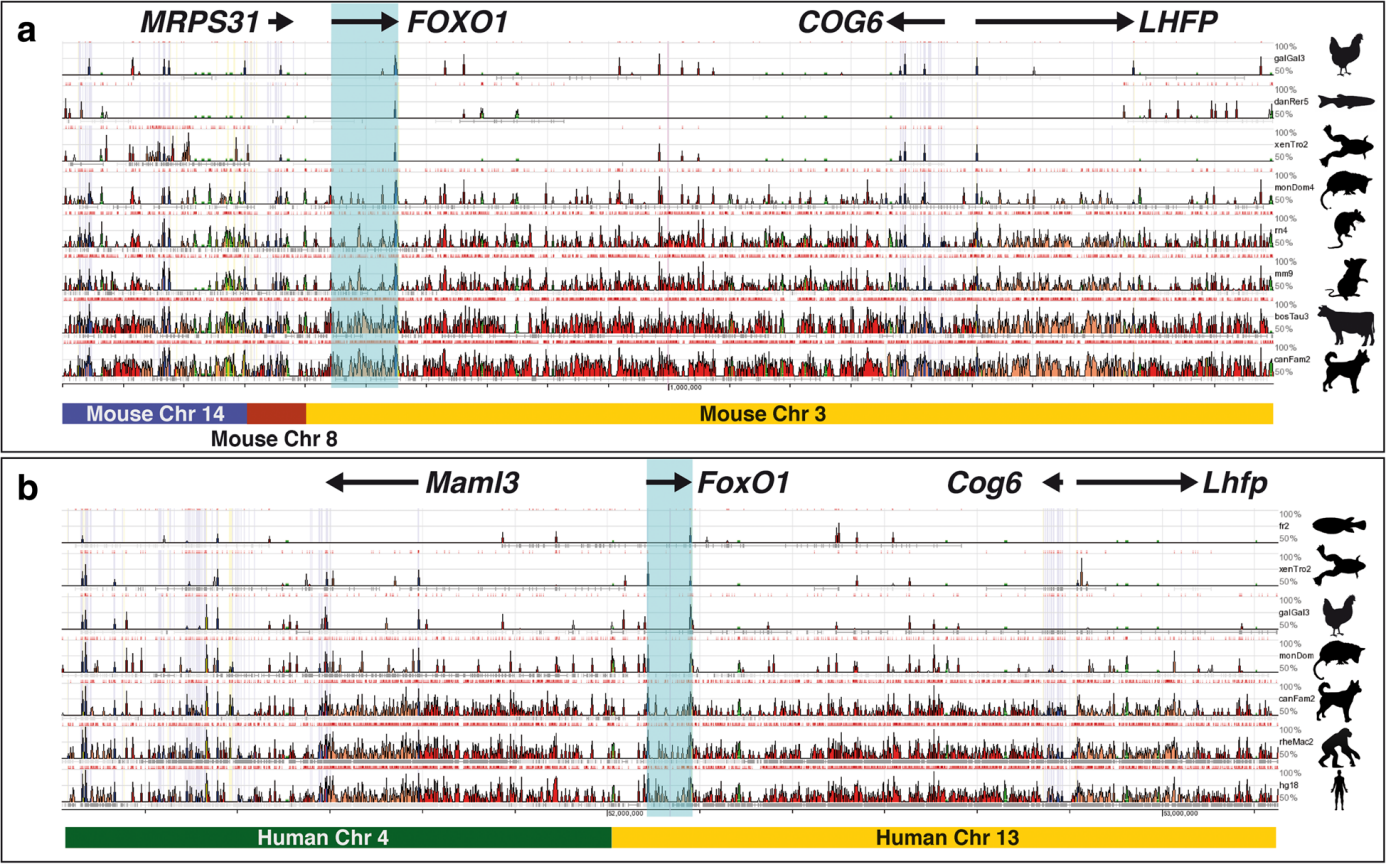
ECRs in the *FOXO1/FoxO1* loci in relation to human–mouse syntenic regions. ECR output from the ECRBrowser tool (http://ecrbrowser.dcode.org, 2015) showing the positions of evolutionary conserved regions when using (**a**) the human and (**b**) mouse genomes as base. Species included in (**a**): Chicken, Zebrafish, Xenopus, Opossum, Rat, Mouse, Cow and Dog; species included in (**b**): Fugu, Xenopus, Chicken, Opossum, Dog, Macaque and Human. Underneath there is a representation of the synteny blocks between human and mouse with indication of the chromosome in which they are found

One of the major unknowns in the study of ARMS is the nature of the cell that originally suffered the *PAX3:FOXO1* chromosomal translocation leading to tumour development. We hypothesised that in the translocated chromosome the fusion gene would be under the control of both *PAX3* and *FOXO1* regulatory elements. For this reason, we first determined the maintenance of synteny surrounding the *FoxO1* locus as an approach to establish the existence of strong constraints on genomic rearrangements as a proxy for the presence of essential *FOXO1* regulatory regions. With the exception of ray-finned fishes, which experienced a whole genome duplication (*D. rerio*, *O. latipes* and *G. aculeatus*; Additional file 1: Figure S1), and rodents (*M. musculus* and *R. rattus*), all species analysed (mammals, birds, amphibians and reptiles) share the same chromosomal structure flanking *FOXO1* (*MRPS31-FOXO1-COG6-LHFP*; Table 1), a structure that has been conserved for at least 450 Mya. The break of synteny upstream of *FoxO1* detected in rodents places the ancestral recombination event in this group between *MRPS31* and *FOXO1* (Fig. 1). Analysis of evolutionarily conserved regions (ECRs) upstream of mouse *FoxO1* shows that a conserved region 47 kb upstream of the gene maps immediately upstream of the human *MAML3* gene on Chr4, while another ECR, located 17 kb upstream of mouse *FoxO1* maps upstream of the human *FOXO1* gene on Chr 13 (Additional file 1: Figure S2). This analysis restricts the ancestral recombination event somewhere in the -17 kb to -47 kb interval upstream of *FOXO1*.

**Table 1:** Location of genes flanking the *FOXO1* locus in human Chr13 across species

	*LHFP*	*COG6*	*FOXO1*	*MRPS31*	
*Homo sapiens*	Chr 13	Chr 13	Chr 13	Chr 13	Human
*Macaca mulatta*	Chr 17	Chr 17	Chr 17	Chr 17	Macaque
*Callithrix jacchus*	Chr 5	Chr 5	Chr 5	Chr 5	Marmoset
*Canis lupus familiaris*	Chr 25	Chr 25	Chr 25	Chr 25	Dog
*Monodelphis domestica*	Chr 4	Chr 4	Chr 4	Chr 4	Opossum
*Mus musculus*	Chr 3	Chr 3	Chr 3	Chr 8	Mouse
*Rattus norvegicus*	Chr 2	Chr 2	Chr 2	Chr 16	Rat
*Gallus gallus*	Chr 1	Chr 1	Chr 1	Chr 1	Chicken
*Alligator mississippiensis*	JH731763	JH731763	JH731763	JH731763	American alligator
*Xenopus tropicalis*	GL172869	GL172869	GL172869	GL172869	Clawed frog
*Latimeria chalumnae*	JH129255	JH129255	JH127414	JH127414	Coalecanth
*Danio rerio*	Chr 10/15	Chr 15	Chr 10/15	Chr 5	Zebrafish
*Oryzias latipes*	Chr 13	Chr 13	Chr 13/14	Chr 14	Medaka
*Gasterosteus aculeatus*	Group I	Group I	Group I/VII	Group VII	Stickleback
*Callorhinchus milli*	KI635872	KI635872	KI635872	KI635872	Elephant shark

Gene names are on the top row, animal species on the left column, common names on the right column. In bold, genes mapping to a different syntenic region. The Coelacanth (*L. chalumnae*) genome is fractioned at present and thus it is not possible to ascertain if the *LHFP/COG6* and *FOXO1/MRPS31* scaffolds are contiguous

In the case of the *Pax3* locus, the same gene organisation was found in all species analysed: *FARSB-SGPP2-PAX3-EPHA4*. Since no breaks in synteny were observed, no conclusions could be drawn on the span of *Pax3* regulatory elements in the locus but it suggests that strong evolutionary constrains have maintained this syntenic block unaltered.

### Hi-C and 4C-seq analyses of the *PAX3/Pax3* and *FOXO1/FoxO1* loci

**Fig. 2:**
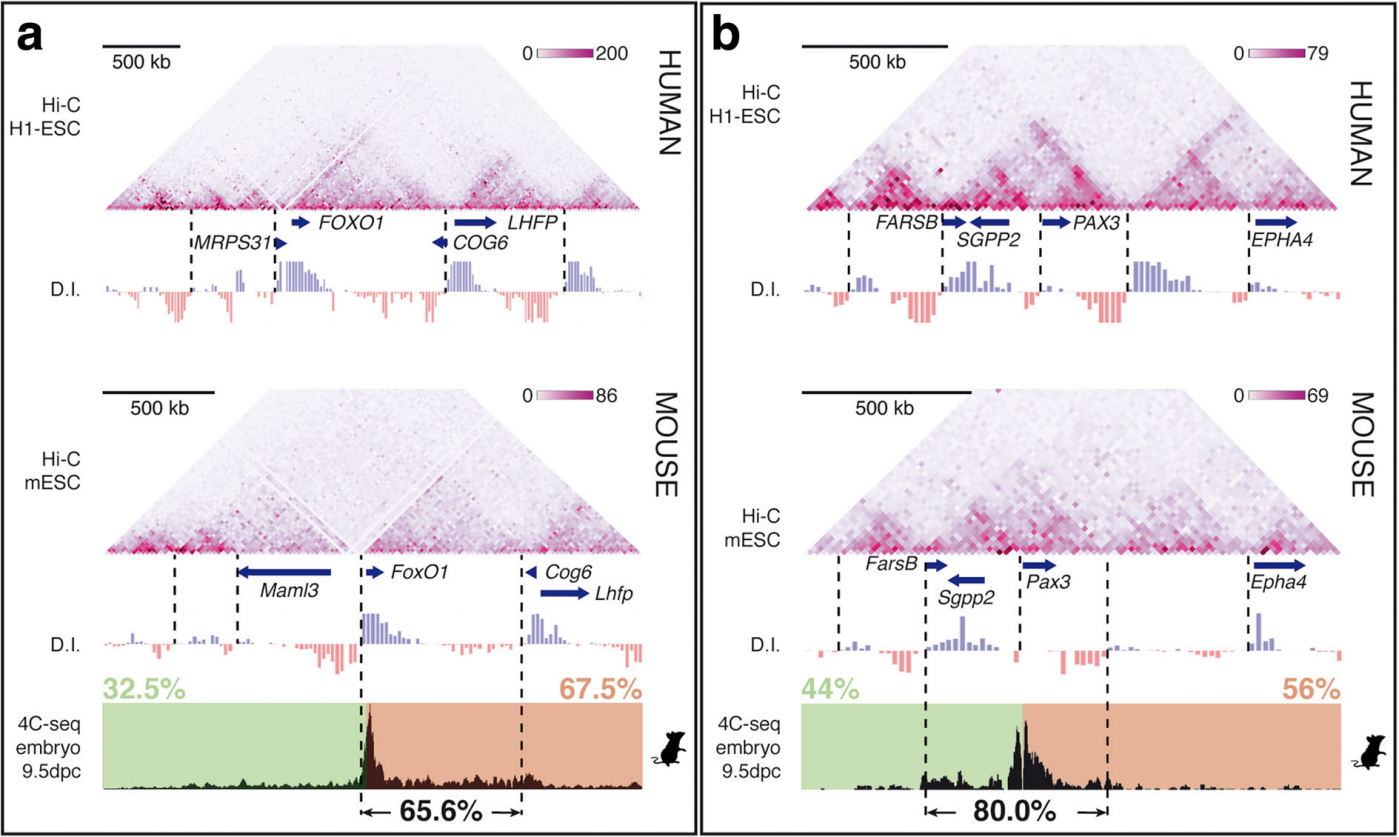
*FoxO1* and *Pax3* promoter interactions are restricted to their respective TADs. (**a**) *FOXO1/FoxO1* and (**b**) *PAX3/Pax3* Hi-C data (2, 5; 20 kb resolution) coincide with the interactions detected by 4C-seq. The size and position of the *FOXO1* and *FoxO1* TADs, as determined by D.I. analyses, are very similar despite the ancestral recombinations that occurred immediately upstream of the genes. Names and positions of the genes in the different loci are indicated. The transcriptional direction of the different genes is indicated. The percentage of accumulated reads 1 Mb at either side of the *FoxO1* and *Pax3* promoters and within the TADs are shown. In the human genome, the positions of the calculated borders for (**a**) the *FOXO1* locus are (*left* to *right*) Chr13: 41,902,000; Chr13: 41,362,000; Chr13: 40,262,000 and Chr13: 39,502,000 and for (**b**) the *PAX3* locus are (*left* to *right*) Chr2: 223,817,756; Chr2: 223,511,756; Chr2: 223,171,756 (sub-TAD boundary); Chr2: 222,871,756 and Chr2: 222,451,756

We then made use of published Hi-C data on human [2] and mouse [5] ES cells, which show that the mouse *FoxO1* gene is included within a single TAD (Fig. 2a), as defined by directionality index analysis (D.I.; 2). Despite the break of synteny immediately upstream of *FOXO1/FoxO1*, the TADs have been maintained in the two species, with similar upstream and downstream borders indicating that the ancestral recombination that gave rise to the synteny break occurred at the TAD border, as shown for other loci [10]. *PAX3/Pax3* are also located in identical TADs in the two species, containing the *SGPP2* and *FARSB* genes and being separated from the *EPHA4* regulatory landscape (Fig. 2b). Our analysis shows the existence of a TAD boundary immediately upstream of *PAX3* in both species. Nevertheless, the Hi-C data reveal extensive contacts between the two domains separated by this putative TAD boundary, suggesting these two domains correspond to sub-TAD structures rather than individual TADs.

We sought to further explore the regulatory landscape for these genes by performing 4C-seq on 9.5 d*pc* (days *post coitum*) whole mouse embryos using the *FoxO1* and *Pax3* promoters as viewpoints. At this developmental stage, both genes are expressed in a variety of progenitor and differentiated cells and thus the 4C-seq data represent an average through different cell types, although overall TAD organisation is mainly invariant across multiple tissues [2, 30]. The data show that interactions of the mouse *Pax3* promoter are almost equally distributed on either side (44% and 56%) and mainly restricted to the TAD that contains it (80.0%), further supporting the hypothesis that the identified boundary immediately upstream of *Pax3* corresponds to a sub-TAD boundary, with *Pax3* regulatory elements being present in both domains. The mouse *FoxO1* promoter interacts preferentially with downstream sequences (67.5%), mainly restricted to the TAD (65.6%); sequences that coincide with H3K27ac active-enhancer marks (Additional file 1: Figure S3) are detected in multiple tissues known to express *FoxO1* [31].

If *FOXO1* enhancer regions are involved in the regulation of the *PAX3:FOXO1* fusion gene, then first we had to gain an insight on the transcriptional regulation of the *FOXO1* gene, identify some of these regions and show that they might be located downstream of the translocation breakpoints.

### Identification of translocation breakpoints in different ARMS cell lines

In ARMS, the t(2;13)(q35;q14) translocation occurs between intron 1 of *FOXO1* and intron 7 of *PAX3* [32–34]. In order to determine the contribution of putative enhancer elements translocated to the derivative t(2;13) chromosome towards the new regulatory landscape, we mapped six independent breakpoints in five independent ARMS cell lines harbouring this translocation. A series of forward primers around 3 kb apart from each other were designed to span the entire *PAX3* intron 7 (18.7 kb) while a series of reverse primers spaced by ~10 kb was designed to span the entire *FOXO1* intron 1 (104.7 kb) (Additional file 2: Table S1). Forward and reverse primers were used in all possible combinations in a long-distance polymerase chain reaction (LD-PCR) designed to amplify fragments up to 20 kb in length.

**Fig. 3:**
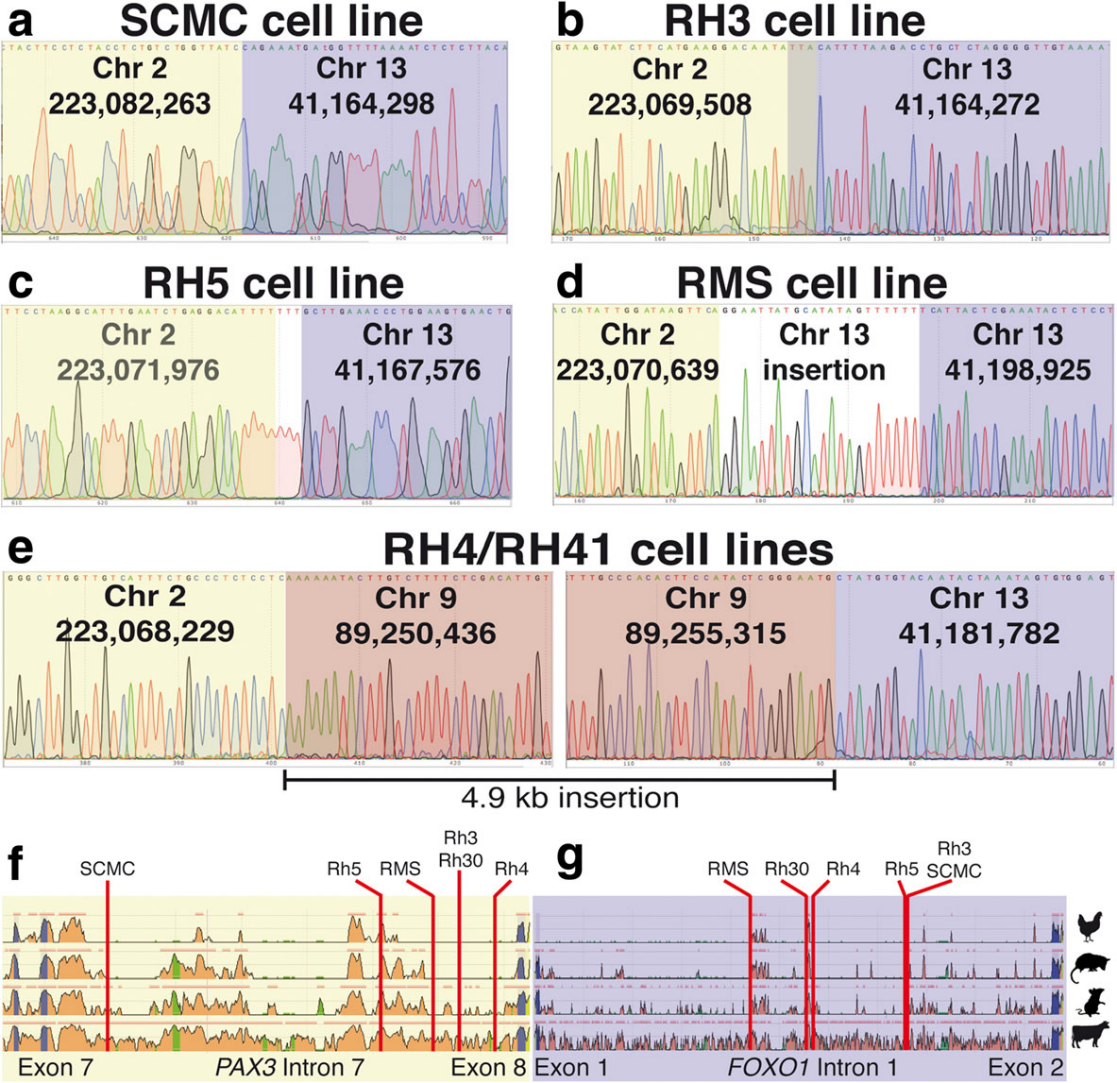
Mapping ARMS translocations to the base pair level. Sequence tracks of the translocation breakpoints identified in five independent ARMS cell lines: (**a**) SCMC, (**b**) RH3, (**c**) RH5, (**d**) RMS and (**e**) RH41. In three cases, the translocation produces a clean cut between Chr2 (*yellow*) and Chr13 (*purple*) sequences. **e** In the RH41 cell line there is a clean insertion of a 4.9 kb fragment from Chr9 (*red*). The genome positions of the translocation breakpoints are provided (hg19). **f** Detail of the ECR Browser output (Chicken, Opossum, Mouse, Cow; base genome, Human) covering the genomic interval between exons 6 and 8 of *PAX3* showing the precise location of the mapped translocation breakpoints in intron 7. **g** Detail of the ECR Browser output covering the genomic interval between exons 1 and 2 of *FOXO1* showing the location of the mapped translocation breakpoints in intron 1

Sequence analyses of the SCMC and RH3 breakpoints showed a seamless transition between *PAX3* and *FOXO1* loci (Fig. 3a, b), although the exact point of the RH3 breakpoint cannot be ascertained as it occurs at a region of micro-homology between the two loci (TTA). The sequence of the RH5 breakpoint (Fig. 3c) showed a small amplification of three thymines at the junction between the *PAX3* and *FOXO1* loci. The RMS breakpoint (Fig. 3d) has a 22 bp insertion of a duplicated fragment from chromosome 13 immediately adjacent to the breakpoint. Finally, cell lines RH4 and RH41, derived from the same patient, show the same breakpoint containing a 4.9 kb insertion from chromosome 9 (Fig. 3e). We have previously reported the identification of the RH30 breakpoint [28].

### Identification of regulatory regions driving transcription of the *FoxO1* and *Pax3* genes

**Fig. 4:**
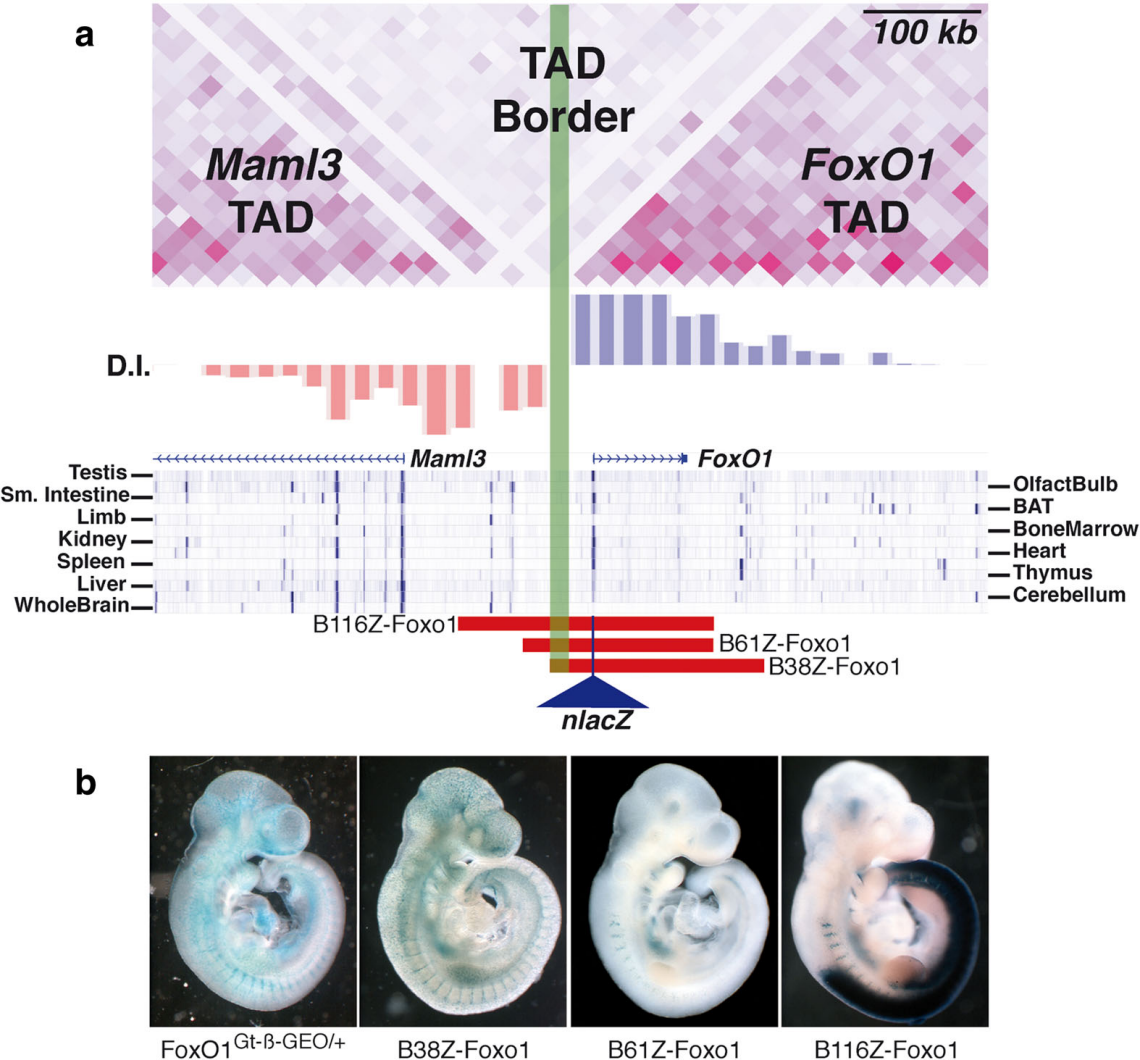
Crossing the TAD border can drive ectopic transgene expression from enhancers in the adjacent domain. **a** Detail of the Hi-C data from mouse ES cells at the TAD border (*green box*). H3K27ac marks in different mouse tissues are shown underneath, as well as the position of the two coding genes in the region and the relative positions of the three BAC clones used in the study. The 5' ends of the clones cross the TAD border, although in the case of B38, its end maps within the border region. There are strong active-enhancer marks in the non-overlapping region between B116 and B61. **b** Expression patterns of *Foxo1* ^Gt-β-GEO/+^, B38Z, B61Z and B116Z at 9.5 d*pc*. B116Z drives strong expression in the CNS, while B61Z does not; B38Z drives strong vascular expression like the gene-trap allele. *D.I.* directionality index

For *FoxO1*, three overlapping bacterial artificial chromosomes (BACs) were selected from the Children’s Hospital Oakland Research Institute (CHORI) library: RP23-66C15 (*-*116 kb to +104 kb, relative to the *FoxO1* transcriptional start site or TSS), RP24-330H17 (*-*61 kb to +104 kb) and RP23-96D10 (-38 kb to +148 kb) (Fig. 4a). We introduced a *lacZ* reporter gene at the first coding ATG of *FoxO1* and renamed them according to the lengths of their upstream spans (B116Z-Foxo1, B61Z-Foxo1 and B38Z-Foxo1, respectively). The 5'-end of B38Z-Foxo1 is located within the interval where the loss of synteny occurs and at the TAD border, while B116Z-Foxo1 and B61Z-Foxo1*,* with almost identical 3'-ends, cross it. We compared the expression patterns driven by these with that of the Foxo1Gt(AD0086)Wtsi gene-trap line (*Foxo1* ^Gt-β-GEO/+^; [31]).

As expected, all of them fail to recapitulate the complete *FoxO1* expression pattern because none of them contains the full regulatory landscape, which our 4C-seq data indicate spans up to 700 kb downstream of the gene. Interestingly, the B116Z-Foxo1 BAC construct drives ectopic expression in the neural tube (Fig. 4b). Unlike B61Z-Foxo1, which also crosses the TAD border, B116Z-Foxo1 contains regions with strong active-enhancer marks in several tissues including some pertaining to the central nervous system. Thus, in this context, the sequence underlying this TAD border does not possess intrinsic transcriptional boundary activity per se because it is unable to block the interactions between regulatory elements and the promoter when placed in between them. Except for this remarkable difference, B61Z-Foxo1 and B116Z-Foxo1 drive very similar expression patterns from 9.5 d*pc* to the adult (note that their 3'-ends are almost identical; compare Additional file 1: Figures S4 and S5). Sites of expression include the myotome, fore-gut and hind-gut diverticula, the stomach, the apical ectodermal ridge (AER), limb, thoracic and facial skeletal muscle, the inner layer of the retina, the posterior wall of the lens vesicle, and the nasal pits. In contrast, the B38Z-Foxo1 construct drives expression from 9.0 d*pc* in vascular precursors throughout the embryo (Fig. 4b and Additional file 1: Figure S6). This finding indicates that a regulatory module for vasculature expression maps in the non-overlapping region between B61Z-Foxo1/B116Z-Foxo1 and B38Z-Foxo1, that is, +104 to +148 kb from the *FoxO1* TSS. Time course analyses of these transgenic lines revealed that all three constructs fail to recapitulate the complete *FoxO1* expression pattern (e.g. no expression is observed in brown adipose tissue -BAT- from 16.5 d*pc* onwards in any of the lines), indicating that the enhancer(s) responsible to drive BAT expression is not contained within these BAC clones.

In order to analyse *Pax3* gene expression, several BAC clones were identified from the CHORI library; for this study we selected RP23-260 F1 (end-sequences GeneBank accession numbers: AQ927932 and AQ927929). This BAC carries 30 kb and 135 kb of sequences upstream and downstream of the transcriptional start point of *Pax3*, respectively (Additional file 1: Figure S7a). Thus, the BAC is completely embedded within the TAD although it crosses the putative sub-TAD border. This BAC was modified by the introduction of a *nlacZ-*SV40pA cassette at the translational start point of *Pax3* (construct B30Z-Pax3) and used to generate transgenic lines. The transgene closely follows the endogenous pattern of *Pax3* [35], being expressed in the neural tube, neural crest cells, somites, the hindbrain, the midbrain and forebrain, migrating limb and hyploglossal chord muscle precursors, the pre-somitic mesoderm, trigeminal ganglia and the lateral nasal process (Additional file 1: Figure S7b).

We generated additional lines using another BAC construct carrying 14 kb upstream of the *Pax3* translational start site and 128 kb downstream of it (RP24-235I14). Analysis of transgenic animals carrying B14Z-Pax3 (Additional file 1: Figure S7c) shows an identical pattern of expression to that driven by the B30Z-Pax3 described above. Therefore, the majority of the regulatory elements needed for the correct spatiotemporal expression of *Pax3* during embryonic development are presumably contained within this BAC.

### Identification of regulatory regions downstream of the RH30 translocation breakpoint

**Fig. 5:**
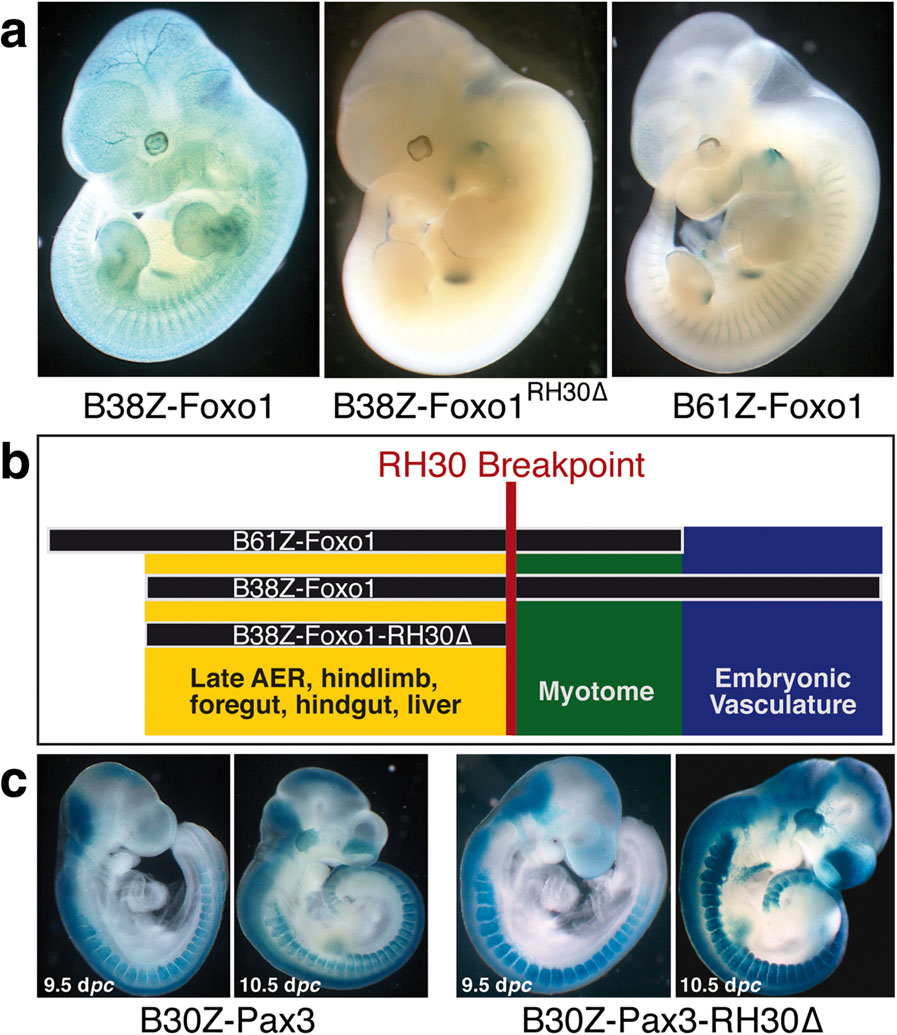
*Pax3* and *FoxO1* enhancers located downstream of the RH30 breakpoint. **a** At 11.5 d*pc*, B38Z-Foxo1 drives expression in the embryonic vasculature, a hindlimb rostral domain, AER, stomach and gut. Myotomal expression is faint and masked by the vascular expression; B61Z-Foxo1 expresses in all these domains excluding the vasculature; B38Z-Foxo1-RH30∆ is not able to drive vascular or myotomal expression. **b** *Map* showing the position of enhancer regions identified downstream of the RH30 translocation breakpoint. The three BAC clones are represented as *black bars* with their given names. Different *coloured boxes* represent the location of different enhancer regions within the BACs. **c** Deletion of sequences downstream the RH30 breakpoint in B30Z-Pax3 do not result in a pattern change

We wanted to examine the enhancer potential of sequences situated downstream of the translocations in ARMS and for this we generated a new BAC construct in which all sequences downstream of the translocation breakpoint found in the RH30 cell line were deleted (B38Z-*Foxo1-*RH30∆). We selected this particular breakpoint because the new regulatory landscape generated by the translocation in the RH30 cell line putatively carries more *PAX3* and *FOXO1* regulatory elements than the other cell lines analysed. Comparison of the expression patterns driven by the B38Z-Foxo1, B61Z-Foxo1 and B38Z-Foxo1*-*RH30∆ (Fig. 5a) in transgenic embryos shows that both the myotomal and embryonic vascular enhancers are located downstream of the RH30 translocation, as B38Z-Foxo1*-*RH30∆ only drives expression in the AER, the foregut and the stomach. This allows the generation of a preliminary map (Fig. 5b) for the location of enhancer elements in relation to the RH30 translocation, which shows that while the enhancer elements driving expression in the developing fore-gut and hind-gut, the stomach and the AER are located upstream of the RH30 translocation, at least two major enhancers are located downstream of this translocation breakpoint. It is also important to highlight other sites of *FoxO1* expression in the mouse (e.g. brown adipose tissue or BAT), not observed in our transgenic lines but detected in a gene trap mouse strain [31], indicating that the regulatory elements controlling the expression at these other sites are not located within the BACs analysed, but further downstream. Thus, in the translocated chromosome, the *PAX3* promoter is in close proximity, at least in the linear genome, to enhancers active in non-*PAX3* territories (e.g. embryonic vasculature and BAT).

Deletion of the sequences downstream of the RH30 translocation breakpoint from B30Z-Pax3 (construct B30Z-Pax3-RH30) has a very limited effect on the overall expression pattern (Fig. 5c), with some changes in intensity levels at some locations. This result suggests that most, if not all, *PAX3* regulatory modules will be carried by the derivative t(2;13)(q35;q14) chromosome following the translocation event.

### Fused regulatory landscape in ARMS

**Fig. 6:**
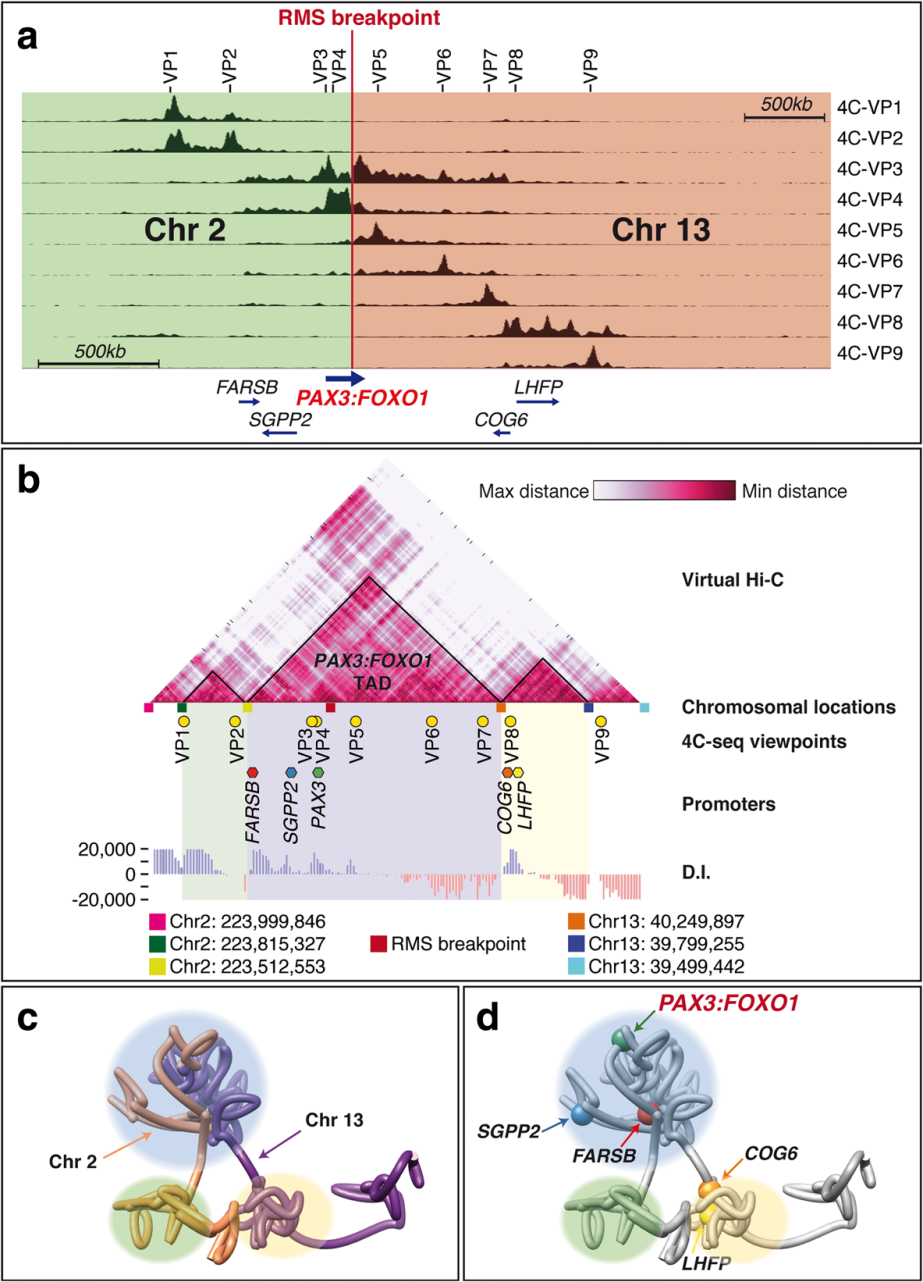
Virtual-HiC of the *PAX3-FOXO1* locus in RMS cells predicts the generation of a new TAD. **a** 4C-seq profiles using nine different viewpoints (VP1–VP9) spanning 3.5 Mb. The locations of the viewpoints are indicated above the *graph*. The location of the fusion *PAX3:FOXO1* gene and other coding sequences is indicated, as well as the position of the RMS breakpoint. *Green* and *orange boxes* indicate reads mapped to Chr2 or Chr13, respectively. **b** The virtual-Hi-C generated from the 4C-seq data was subjected to a D.I. analysis to determine the location of TAD borders. The upstream and downstream borders thus defined closely match those obtained by D.I. analyses of human Hi-C data while a novel TAD encompassing the *PAX3-FOXO1* fusion locus is predicted. The positions of the viewpoints (*pale green circles*), the promoters of the genes in the region (*coloured hexagons*) and the borders identified by D.I. analysis (*coloured boxes*) are indicated. The chromosomal coordinates of the predicted borders are provided underneath. 3D chromatin architecture model for the locus encompassing the translocation in ARMS, (**c**) showing the contribution of both chromosome regions to the predicted new TAD and (**d**) the location of promoter sequences within the TAD

We hypothesised that the translocation event would generate a fusion of the regulatory landscapes defined by the upstream and downstream boundaries of *PAX3* and *FOXO1*, respectively (Fig. 2). This new regulatory landscape would therefore allow the interaction of the *PAX3* promoter with *FOXO1* regulatory sequences and drive the expression of the oncogene in non-*PAX3* territories. To test this, we performed 4C-seq using chromatin from the patient-derived cell line RMS taking viewpoints scattered throughout the *PAX3:FOXO1* fused locus (Fig. 6a). Some of them correspond to CTCF binding sites (VP1, VP2, VP6, VP8 and VP9), while others coincide with ECRs (VP4, VP5, VP7). Specifically, VP4 marks a well-known *PAX3* enhancer that drives neural crest expression [36]. Functional activity of the other two ECRs has not been determined, but they are enriched in active chromatin marks in various tissues. VP3 corresponds to the *PAX3* promoter. 4C-seq data were integrated to create virtual 3D chromatin conformation models (Additional file 3: Movie S1), which were further converted into a virtual Hi-C heatmap (Fig. 6b), as previously described [37]. As an example, one of the virtual models generated is represented in Fig. 6c and d and Additional file 4: Movie S2.

As predicted, the chromosomal rearrangement that takes place in RMS cells generates a new TAD as the result of the fusion of *PAX3* and *FOXO1* regulatory landscapes. Importantly, the borders of this new TAD coincide with those calculated in the wild-type loci (compare the positions of the borders in Figs. 2 and 6). Furthermore, these translocation TAD borders are mainly invariant across a multitude of human tissues (Additional file 2: Table S2), the upstream *PAX3* border and the downstream *FOXO1* border being conserved at a +/– 20 kb resolution in 61.9% and 66.7% of the 21 cell types/tissues analysed, respectively [30]. Thus, the new TAD harbours the *PAX3:FOXO1* fusion gene, as well as *FARSB* and *SGPP2*, while the flanking TADs remain mainly unchanged, with the exception of the boundary at the end of the analysed region, which shows a significant difference. Nevertheless, as this particular predicted boundary is at the end of the analysed region, it may arise as an artefact of the computational approach, which is not reliable at the extremes. Interestingly, the 4C-seq data indicate that these flanking TADs interact with each other (note the rhomboid-like domain above the *PAX3-FOXO1* TAD in Fig. 6b), presumably reinforcing the formation of an isolated highly self-interacting domain in between them. Although the D.I. analysis of the virtual Hi-C data does not reveal the existence of the predicted sub-TAD containing *SGPP2* (as observed in the Hi-C analyses of wild-type mouse and human loci), the 3D chromatin structure model clearly shows an isolated chromosomal loop that contains the *SGPP2* promoter (Fig. 6d and Additional file 3: Movie S1; Additional file 4: Movie S2).

### The human *PAX3* promoter is able to interact with potential *FOXO1* enhancers in RMS cells

**Fig. 7:**
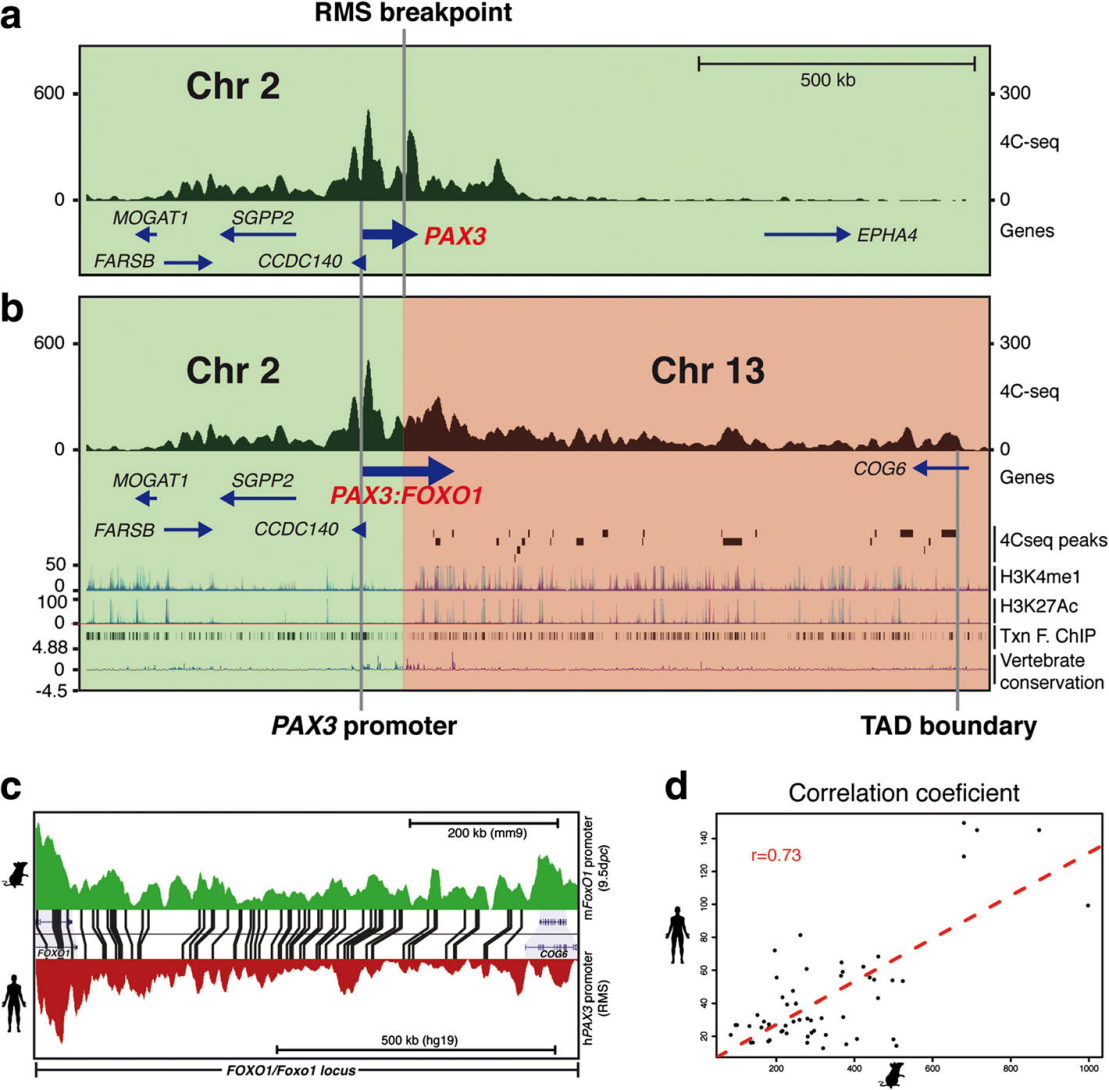
The *PAX3* promoter interacts with *FOXO1* sequences in a patient-derived ARMS cell line. 4C-seq profiles on (**a**) the *PAX3* and (**b**) the *PAX3-FOXO1* loci obtained using the *PAX3* promoter as a viewpoint in the RMS cell line. The locations of the promoter and the translocation breakpoint in are indicated. In (**b**), the *first row* represents the derivative t(2:13) chromosome 4C-seq profile. *Green* and *orange boxes* indicate reads mapped to Chr2 or Chr13, respectively. The *second row* shows the location of the fusion *PAX3:FOXO1* gene and other coding sequences. The *third row* indicates the locations of 4C-peaks defined using the Peak Calling algorithm; the downstream limit was taken as the TAD boundary defined in the previous experiment. The *fourth* and *fifth rows* show H3K4me1 and H3K27ac marks in different tissues, respectively. The *sixth row* is the transcription factor-ChIP track from UCSC. The *seventh row* indicates vertebrate conservation. **c** Sequence-paired 4C-seq tracks on the *FOXO1/FoxO1* locus from mouse embryos (*green*) and the RMS cell line (*red*) showing the location of ECRs shared between human, mouse and opossum genomes as shown in Additional file 1: Figure S3. **d** Correlation between the 4C-seq signal in the *FOXO1/FoxO1* loci from mouse embryos and RMS human cells. For each conserved element, the 4C-seq signal in human cells corresponding to this region is plotted against the 4C-seq signal from mouse embryos in the orthologous region. The *red dashed line* represents the linear regression line

Having demonstrated that the *PAX3* promoter lies in the same domain as *FOXO1* regulatory elements in the translocated chromosome, we sought to determine if, indeed, they could interact with each other to drive the expression of the oncogene in *FOXO1*-specific tissues. For this reason, we focused on the 4C-seq data that take the human *PAX3* promoter as a viewpoint and detected strong interactions between the *PAX3* promoter and *FOXO1* regions situated downstream of the identified breakpoint in the RMS cell line (Fig. 7). The first ectopic contacts on the *FOXO1* locus occur immediately downstream of the defined breakpoint, strengthening further our breakpoint mapping strategy. Furthermore, the span and location of the interactions of the *PAX3* promoter with the *FOXO1* locus in the translocation closely match those detected by 4C-seq in the mouse locus (Fig. 7c and d), suggesting that the *FOXO1* region within the novel TAD is folded in a structure similar to that of the wild-type *FOXO1* locus in chromosome 13; it is within this new chromatin structure that interactions between *FOXO1* regulatory elements and the *PAX3* promoter take place. We then applied a peak-calling algorithm that was able to detect 24 interaction peaks from the translocation point to the TAD border (Additional file 1: Figure S8 and Additional file 2: Table S3). Many of these peaks (16/24) are enriched in active chromatin marks in a variety of tissues known to express *FOXO1*, including skeletal muscle, adipose nuclei and endothelial cells. Also, some of them contain ECRs (5/24), as well as experimentally validated (ChIP-seq) binding sites (14/24) for specific transcription factors (e.g. EP300, MEF2A or CEBPB) or structural proteins such as CTCF and RAD21 (9/24). Together, these data suggest that the *PAX3* promoter engages in interactions with potential *FOXO1* regulatory elements in the translocated chromosome in ARMS tumours, interactions that are restricted to the wild-type 3' TAD border of the *FOXO1* locus.

## Discussion

### Transcriptional regulation of *FOXO1* and *PAX3*

The transgenic analyses show that *FoxO1* is regulated by individual regulatory regions driving expression of the transgene in different anatomical locations during embryonic development and in the adult. Importantly, we have mapped the enhancer responsible for embryonic vascular expression to the non-overlapping region between B61Z and B38Z (the +104 kb to +148 kb interval), downstream of exon 2 and thus located downstream of all translocation breakpoints in ARMS. None of our constructs is able to direct expression in brown adipose tissue (BAT), a strong site of expression for the endogenous *FoxO1* [31], indicating that this element is located further downstream.

In the case of *Pax3*, differences in the relative intensity of expression between neural tube and somites probably arise from the perdurance of β-galactosidase activity, as noted for other *lacZ* transgenes [38], and the existence of a micro RNA sequence in the 3'UTR of *Pax3* [39, 40] that downregulates somitic expression but cannot act on our *lacZ* construct as it is terminated by the SV40pA sequence. The fact that B14Z-Pax3 contains the 14 kb interval previously described [41] as the only required sequences upstream of the *Pax3* gene and that it can drive most, if not all, of the *Pax3* endogenous pattern during early embryonic development, suggests that most of the embryonic *Pax3* regulatory elements are located downstream of the *Pax3* translational start site.

### Structural organisation of the *PAX3:FOXO1* locus in ARMS

Our synteny analysis shows that the chromosomal structure that includes the *FOXO1* locus (*LHFP-COG6-FOXO1-MRPS31*) is highly conserved between species as evolutionary distant as the cartilaginous fish Elephant shark (*Callorhinchus milii*) and humans, revealing that the same gene structure flanking the *FOXO1* gene has been maintained at least over the past 420 Mya. We propose that the localisation of the *FOXO1* promoter in close proximity to the upstream TAD border has been the driving force for the invariant structure of that border. Indeed, a single break of synteny could be identified in all the species covered by our analysis and that arose following a chromosomal rearrangement at the base of the rodents precisely at the interface between the two TAD structures.

Other changes in the genomes of teleosts also took place following the whole genome duplication event at the base of the bony fish group following chromosomal rearrangements. In the three cases analysed, the structure of the syntenic region has also been maintained and the *FOXO1-COG6-LHFP* syntenic group retained. Interestingly, the original upstream structure has also remained on the paralogous gene, indicating the presence of strong constraints for the disaggregation of these genes and their regulatory sequences, even if duplicated.

The study of oncogenic recurrent chromosomal translocations allows investigation of the effects of chromosomal rearrangements on gene expression without the need to resort to the reconstruction of the effect of evolutionary forces upon the process.

We have shown that in ARMS, the *PAX3* promoter interacts strongly with sequences in the *FOXO1* locus, sequences and interactions that are conserved in the wild-type mouse locus and that, in many cases, correlate with the presence of H3K27Ac marks, DNAseI hypersensitive sites, the binding of diverse transcription factors, and ECRs, indicative of active enhancers. This implies that the *PAX3:FOXO1* oncogene is, at least in part, under the control of *FOXO1* regulatory elements. Furthermore, the profile of interactions between the *PAX3* promoter and *FOXO1* sequences correlates with the profile of interactions observed between the mouse *FoxO1* promoter and its regulatory landscape.

The chromatin extrusion model of TAD formation [42, 43] may explain how the borders flanking the fused TAD are conserved after the translocation. According to this model, loop-extruding factors (likely, cohesins) would load randomly onto the DNA forming a small chromatin loop. Then, these factors would slide through the chromatin in opposite directions while still tethered, progressively extruding the DNA between them creating a larger loop. Once they encounter a boundary element (likely, CTCF in a specific orientation), they would be stalled. The new TAD would thus be formed by the interaction between the pre-existing borders creating a new regulatory landscape in which contacts between the *PAX3* promoter and regulatory elements of *FOXO1* take place*.* We cannot exclude that these interactions may contribute to the formation and/or maintenance of the new TAD, as previously suggested in the case of the *Xist* locus [44].

TADs are composed of and are a consequence of chromatin interactions. However, in the case of the *PAX3:FOXO1* TAD we argue against a model in which TAD formation is caused by the pre-establishment of specific enhancer-promoter or enhancer-enhancer regulatory interactions. The translocation places the *PAX3* promoter and enhancers from both genes in a new regulatory environment. We would argue that in this new environment the interactions would be significantly different from those established in the wild-type locus and thus if these preceded TAD organisation, a shift of the position of the borders would have been observed.

It has recently been reported that active transcription or gene looping is not required for TAD formation [45]. The authors show conservation of TAD organisation around the *CFTR* locus in five different cancer cell lines, two of which do not express the gene. Furthermore, looping interactions within the *CFTR*-containing TAD (intra-TAD interactions) were highly specific in those cells that express the gene and absent in those that do not express it. Thus, as previously reported [2, 46], internal TAD organisation is cell-type specific whereas overall TAD structures are mostly conserved, which argues against a model in which TADs are passively formed as a consequence of the establishment of specific regulatory interactions. Additionally, such a model in which the emphasis is placed on the interactions and not on the importance of a border would not explain why the removal of TAD boundaries cause adjacent TADs to merge and a rewiring of regulatory interactions [17–19].

Our analyses also show that while both B61Z- and B116Z-Foxo1 cross the *FoxO1* 5'-TAD border, only B116Z-Foxo1 spans into regions marked by H3K27ac in the whole brain, cerebellum and olfactory tract, which suggest the presence of active neural tissue enhancers. Therefore, the sequence of this TAD border is not sufficient to separate regulatory landscapes, indicating that efficient separation may require interaction between TAD-border sequences, such as convergent CTCF binding sites [15, 16], and other sequences within the TAD domains. In fact, close observation of the mouse Hi-C data reveals that the borders of the *FoxO1*-containing TAD do interact with each other (note the interactions at the peak of the triangle depicting the third TAD at the bottom of Fig. 2a).

### Implications for the cell type of origin for ARMS

ARMS tumours appear generally in trunk and extremities [47], but examples of other sites of primary ARMS abound in the literature (e.g. [48–53]), suggesting that they can arise in multiple cell types or in a single cell type found throughout the body, with certain locations such as the extremities being more susceptible than others. ARMS tumours are characterised by the expression of muscle-specific markers (reviewed in [54]), suggesting a possible myogenic origin, although their molecular characteristics are more related to cells that have been committed to the myogenic lineage but are unable to complete terminal differentiation to become skeletal muscle. For example, it has been shown that *MYOD* is activated by the *PAX3-FOXO1* fusion protein while it interferes with its chromatin remodelling functions, inhibiting the expression of the skeletal muscle terminal differentiation factor, *MYOG* [55]. An interesting hypothesis is that dysregulation of PAX3 or PAX7 target genes may result in the activation of the myogenic programme in a non-myogenic lineage, the cells being able to transdifferentiate but unable to fully complete terminal differentiation. It has been shown that ectopic expression of *PAX3* in the lateral plate mesoderm of chick embryos induces the expression of the myogenic regulatory factors *MYF5*, *MYOG* and *MYOD* [56]; expression in mesenchymal stem cells also induces the activation of myogenic markers such as *MYF5*, *MYOD*, *MYOG*, *MCK* and *MHC*, pushing them towards the myogenic lineage, while blocking their osteogenic, chondrogenic or adipogenic potential [57]. It is thus likely that the myogenic-like transcriptome of ARMS tumours [58] is the result of *PAX3:FOXO1* activation rather than a remnant of their lineage origin.

Several cell types have been previously suggested as the origin for ARMS, corresponding to embryonic, postnatal or adult stem cells or adult myofibres [59], both from the myogenic lineage [60–64] or other lineages [65, 66].

Our data reveal a clear set of interactions in the embryo between the *FoxO1* promoter and, in the RMS cell line, the *PAX3* promoter, and far-downstream sequences in the *FOXO1/FoxO1* locus, which presumably correspond to enhancer regions of the gene.

An interesting site of *FoxO1* expression is BAT [31], which can easily transdifferentiate into muscle and vice versa [67–70], while overexpression of a constitutively active *Smoothened* restricted to adipocytes has been shown to give rise to embryonic rhabdomyosarcomas (ERMS) [71] with relative high penetrance.

None of our constructs drive expression in BAT, indicating that the enhancer(s) responsible for this aspect of the expression is located even further downstream. Indeed, epigenetic marks in BAT from 24-week-old mice indicate active sites coincident with downstream regions that interact strongly with both the mouse *FoxO1* and human *PAX3* promoters (Additional file 1: Figure S3), while our data clearly show that the enhancers required for both embryonic and adult vasculature expression are located downstream of all the mapped translocation breakpoints.

Another important site of expression is the developing and adult vasculature, although we have not identified the different cell types associated with this expression. In the embryo, some progenitors for vasculature and skeletal muscle reside in the dermomyotome and their fate decision depends on the ratio between *Pax3* and *Foxc2*, acting as pro-myogenic and pro-angiogenic factors, respectively. Importantly, *Foxc2* expression is repressed both by PAX3 and the PAX3-FOXO1 fusion protein, promoting myogenesis in cells that, under normal circumstances, would not give rise to skeletal muscle [72]. Therefore, we propose the BAT and vasculature cell lineages as new candidates for the cell type of origin for ARMS. As the survival rates for these types of tumour are particularly low (around 70% of patients show recurrent tumour resurgence following current therapies), the final identification of the lineages that can serve as origin for ARMS will provide further information on the biology of these tumours and the importance of additional activating mutations specific for each lineage, opening new avenues for the development of new targeted therapies based on the transcriptome and epigenome of the individual cell types of origin.

## Conclusions

We have shown that novel regulatory landscapes arise as a result of oncogenic human translocations and that these are restricted by the original upstream and downstream TAD boundaries of the genes involved in the translocation, indicating that TAD formation precedes intra-TAD interactions. We have identified several major enhancer regions for *FOXO1* present downstream of all t(2;13) translocations in ARMS and thus potentially able to drive expression of the oncogene in non-*PAX3*-expressing cells. We also indicate that brown adipose tissue and the vasculature should be considered in future studies on cell lineage of origin for ARMS. Ectopic oncogene activation may be an essential step in the tumorigenic process, as expression in a particular cell type, the often-elusive cell of origin, may be required for disease development.

## Methods

### Integration of a *LacZ* reporter gene into BAC clones

To target the *FoxO1* BACs, homology arms were synthesised by standard PCR methods using the oligonucleotide primers pFoxHAF + *Apa*I/pFoxHAR + *Apa*I (Additional file 2: Table S1) which generate a 410 bp fragment spanning 204 bp and 206 bp upstream and downstream of the first coding ATG of *FoxO1*, respectively. We then used the single *Nco*I site at position -1 to insert a linker sequence (Additional file 2: Table S1). Into the single *Bgl*II of the linker we then cloned a *galK* selectable marker [73] or a ~3 kb *Bam*HI fragment from our standard construct #1 [74] containing a nuclear-localised *lacZ* reporter gene and a SV40 polyadenylation signal. To target the *Pax3* BACs, homology arms were synthesised by standard PCR methods using the oligonucleotide primer pairs pPax3_5HAF + *Eag*I/pPax3_5HAR + Link and pPax3_3HAF + Link/pPax3_3HAR + *Eag*I (Additional file 2: Table S1) and then joined by PCR. This generates a 950 bp fragment spanning 461 bp and 468 bp upstream and downstream of the first coding ATG of *Pax3*, respectively, and introduces a small polylinker immediately upstream of the gene. We then used the single *Bgl*II site at position -1 to insert the *galK* selectable marker or the nuclear-localised *lacZ* reporter gene and a SV40-polyA. These constituted the targeting cassettes. The B116-Foxo1, B61Z-Foxo1, B38Z-Foxo1, B14-Pax3 and B30-Pax3 BAC constructs were then modified by two-step *galK* recombineering [73] with modifications as previously described [75]. All positive clones were checked for integrity by multiple restriction digests and inserts sequenced prior to pronuclear injection. The number of independent transgenic lines showing similar expression patterns for each construct is as follows: B38Z-Foxo1: four lines; B61Z-Foxo1: three lines; B116Z-Foxo1: three lines; B14Z-Pax3: two lines; B30Z-Pax3: four lines.

### RH30 deletion in BAC clones

To generate the deletions at the RH30 breakpoint sequence in mouse BACs, we made homology cassettes (Additional file 2: Table S1) with ~75 bp of homology at either side of the mouse sequence corresponding to the breakpoint in the RH30 cell line and containing a LoxP511 site in the same orientation as the one in the BAC vector-backbone (pBACe3.6). The cassettes were then inserted by single-step recombineering [73] in B38Z-Foxo1 and B30Z-Pax3. Positive clones were sequenced and transferred into the SW106 *E. coli* bacterial strain [73] that carries an Arabinose-inducible *Cre* gene for the excision of the intervening fragments. Following induction of *Cre* expression, positive clones were identified and checked for integrity by multiple restriction digests; deletions were confirmed by sequencing prior to pronuclear injection. The number of independent transgenic lines showing similar expression patterns for each construct is as follows: B38Z-Foxo1-RH30∆: three lines; B30Z-Pax3-RH30∆: two lines.

### Generation of transgenic mice and embryo analyses

BAC DNA was prepared using the QIAgen maxiprep kit (QIAGEN Ltd., UK) as previously described [75]. After dialysis against microinjection buffer (10 mM Tris-HCl pH 7.5, 0.1 mM EDTA pH 8.0 and 100 mM NaCl), DNA was diluted to 1.6–1.8 ng/mL in microinjection buffer and used for pronuclear injection of fertilised mouse eggs from B6CBAF1/OlaHsd crosses using standard techniques. Embryo β-galactosidase staining was performed as previously described [75]. Embryo pictures were obtained using a Nikon SMZ1500 microscope and a JVC KY-F55B 3-CCD camera connected to a Scion Series 7 card. Images were imported into AdobePhotoshop (v12.0 x64) and whole image correction applied using the ‘AutoLevels’ tool.

### Identification of breakpoints in ARMS cell lines

The RH3, RH28 and RH41 cell lines were obtained from Dr Peter Houghton (St Jude Children’s Research Hospital, Memphis, TN, USA); the RMS, SCMC and RH30 cell lines were a kind gift from Dr Janet Shipley (The Institute of Cancer Research, Sutton, UK). Cells were grown in Dulbecco’s Modified Eagle’s Medium (DMEM, SIGMA UK) supplemented with 10% (v/v) fetal calf serum, 60 mg/mL Benzylpenicillin and 100 mg/mL Streptomycin sulphate. Cells were isolated from two 75 cm^2^ flasks (Nunc) at 80% confluency by standard methods and genomic DNA extracted as previously described [76]. LD-PCR was used to amplify the genomic DNA from the different cell lines using all possible combinations from 11 oligonucleotides evenly spaced over ~110 kb and covering intron 1 of *FOXO1* (Foxo1-LD primers) and seven oligonucleotides evenly spaced over ~27 kb and covering intron 7 of *PAX3* (Pax3-LD primers) (Additional file 2: Table S1). LD-PCR was performed using the Expand Long Template PCR kit (Roche), using Buffer 3, as instructed by the manufacturers. The SCMC breakpoint was amplified with the Foxo1-LD8/Pax3-LD6 primer pair (3.1 kb); the RH3 breakpoint was amplified with the Foxo1-LD8/Pax3-LD2 primer pair (1.3 kb fragment); the RH5 breakpoint was amplified using the Foxo1-LD8/Pax3-LD3 primer pair (5.3 kb); the RMS breakpoint was amplified using the Foxo1-LD5/Pax3-LD3 primer pair (7.8 kb); the RH4/RH41 breakpoint was amplified using the Foxo1-LD7/Pax3-LD3 primer pair (12.8 kb fragment). Products were cloned into pCR2.1-TOPO (Invitrogen) and sequenced. We have previously reported the sequence of the RH30 translocation breakpoint [28].

### 4C-seq analyses

4C-seq assays were performed as previously reported [77–80]. Briefly, hybrid CBA/C57Bl6 mouse embryos at the desired stage were disrupted using 1X PBS/0.125% (w/v) collagenase (Sigma-Aldrich). 10^7^ individual cells were fixed in 1X PBS/2% (w/v) formaldehyde for 15 min at room temperature. A total of 155 μl of 10% (w/v) Glycine were added to stop the fixation, followed by a wash by centrifugation with 1X PBS at 4 °C. Pellets were frozen in liquid nitrogen and kept at -80 °C. Isolated cells were lysed (lysis buffer: 10 mM Tris-HCl pH 8, 10 mM NaCl, 0.3% (v/v) IGEPAL CA-630 [Sigma-Aldrich]), 1X protease inhibitor cocktail (cOmplete, Roche) was added and the DNA digested with *Dpn*II and *Csp*6I as primary and secondary enzymes, respectively. T4 DNA ligase was used for both ligation steps. Specific primers were designed at the genes promoters 4C-mPax3 (mouse *Pax3* promoter), 4C-hPAX3 (human *PAX3* promoter) and 4C-mFoxo1 (mouse *FoxO1* promoter), as well as for the rest of the viewpoints (VP1–VP9) (Additional file 2: Table S1) with Primer3 (v. 0.4.0) [81]. Illumina adaptors were included in the primer sequences. Eight separate PCRs were performed for each viewpoint with Expand Long Template PCR System (Roche) and pooled together. The libraries were purified with a High Pure PCR Product Purification Kit (Roche), concentrations measured using the Quanti-iTTM PicoGreen dsDNA Assay Kit (Invitrogen) and sent for deep sequencing.

### 4C-seq data analyses and 3D chromatin modelling

4C-seq data were analysed as previously described [79]. Briefly, raw sequencing data were de-multiplexed and aligned using mouse July 2007 assembly (mm9) or human February 2009 (hg19) as the reference genomes. Reads located in fragments flanked by two restriction sites of the same enzyme, or in fragments smaller than 40 bp, were filtered out. Mapped reads were then converted to reads-per-first-enzyme-fragment-end units and smoothed using a 30 fragment mean running window algorithm, uploaded to the UCSC genome browser [82] (http://genome.ucsc.edu/, 2015) and subjected to a five-pixel smoothing window. In Fig. 7, as reads upstream of the breakpoint come from both the intact and translocated *PAX3* locus and downstream reads map to *PAX3* or *FOXO1*, 4C-seq scales have been adjusted to normalise reads at either side of the translocation.

The protocol of the chromatin modelling based on 4C-seq data was applied as previously described [37]. Briefly, 4C-seq data were used as a proxy of distance between individual viewpoints and the rest of the DNA fragments under the assumption that 4C-seq reads are inversely proportional to their spatial distance. These distances were used as restraint coordinates to locate the position of DNA fragments in the 3D space. The Integrative Modelling Platform (IMP) [83] was used for the generation of chromatin 3D models. The 200 top-scoring models were selected out of 50,000 and then clustered in two populations that were mirror image of each other. The most populated cluster was selected and used for the calculation of the Virtual Hi-C, as previously described [37].

4C-seq reads corresponding to the derivative t(2:13) chromosome were duplicated in order to compensate the theoretical quantity of whole chromosomes depending on the viewpoint used. Reads were then normalised and the Z-scores calculated as previously described [37] to filter out the non-significant data. For peak calling of 4C-seq data, interaction calling was carried out using as a background a two-sided monotonic regression calculated using the Pool Adjacent Violators Algorithm (PAVA) from the R-package isotone [84]. With this background, we computed the distribution of residuals (differences between observed and expected values for each fragment) and defined as peaks those fragments with residuals that were above the third quartile plus 1.5 × IQR, IQR being the interquartile range [85]. Peaks less than 500 bp apart were merged together in a single unit.

### Directionality index and boundary calling

Boundary calling was carried out using the D.I. [2]. The D.I. at each position is based on fragments contacts for both sides, but we only used data limited to these regions of interest. Thus, we are missing data for the fragments located at the borders. We simulated the missing data for the fragments in the borders by taking the mean value of the complete dataset as reference. We calculated the D.I. of the Hi-C’s for both loci in the two species iteratively, changing the expected TAD size variable in each iteration (Additional file 2: Table S4). We selected the boundaries that appeared in all the iterations. We used the same approach for the virtual Hi-C of the truncated locus but we selected the top two boundaries which appeared in 96% of the iterations (Additional file 2: Table S4). Hi-C data were taken from the Epigenome Browser (http://egg.wustl.edu/d/; 2016); the datasets used for these calculations were: MM9: Esc_20kb_hindIII_rep1_mouse and HG19: Esc_20kb_hindIII_rep2_human.

## Additional files

Additional file 1: Figure S1. Orthologous pairwise clusters involving the *FoxO1* gene. **Figure S2.** Conservation analysis across the *FoxO1-Maml3* intergenic region. **Figure S3.** ECRs identified in the *FoxO1* region downstream of the RMS breakpoint and associated H3K27ac marks. **Figure S4.** Time-course of embryos carrying the B116Z-Foxo1 reporter construct. **Figure S5.** Time-course of embryos carrying the B61Z-Foxo1 reporter construct. **Figure S6.** Time-course of embryos carrying the B38Z-Foxo1 reporter construct. **Figure S7.** Recapitulation of Pax3 endogenous expression pattern by a BAC carrying 30 kb of upstream sequences. **Figure S8** Peaks of interaction established by the *PAX3* promoter at the *FOXO1* locus in RMS cells. (PDF 3020 kb)

Additional file 2: Table S1. Oligonucleotides used in this work. **Table S2.** Comparison of the TAD borders called at the *PAX3* and *FOXO1* human loci. **Table S3.** Interaction peaks between the *PAX3* promoter and regions within the *FOXO1* locus in RMS cells. **Table S4.** Number of times the defined TAD boundaries appeared in the iteration. (PDF 296 kb)

Additional file 3: Movie S1. PAX3:FOXO1 3D superposition model. (MP4 75352 kb)

Additional file 4: Movie S2. PAX3:FOXO1 3D chromatin model. (MP4 9598 kb)
