# Supplementary material for: Regulatory landscape fusion in rhabdomyosarcoma through interactions between the PAX3 promoter and FOXO1 regulatory elements
Source: Genome Biol. 2017 Jun 14;18:106. doi: 10.1186/s13059-017-1225-z (PMC5470208; doi:10.1186/s13059-017-1225-z)
Supplement: Supplementary file 1 — Orthologous pairwise clusters involving the FoxO1 gene. Figure S2. Conservation analysis across the FoxO1-Maml3 intergenic region. Figure S3. ECRs identified in the FoxO1 region downstream of the RMS breakpoint and associated H3K27ac marks. Figure S4. Time-course of embryos carrying the B116Z-Foxo1 reporter construct. Figure S5. Time-course of embryos carrying the B61Z-Foxo1 reporter construct. Figure S6. Time-course of embryos carrying the B38Z-Foxo1 reporter construct. Figure S7. Recapitulation of Pax3 endogenous expression pattern by a BAC carrying 30 kb of upstream sequences. Figure S8 Peaks of interaction established by the PAX3 promoter at the FOXO1 locus in RMS cells. (PDF 3020 kb) [file 13059_2017_1225_MOESM1_ESM.pdf]

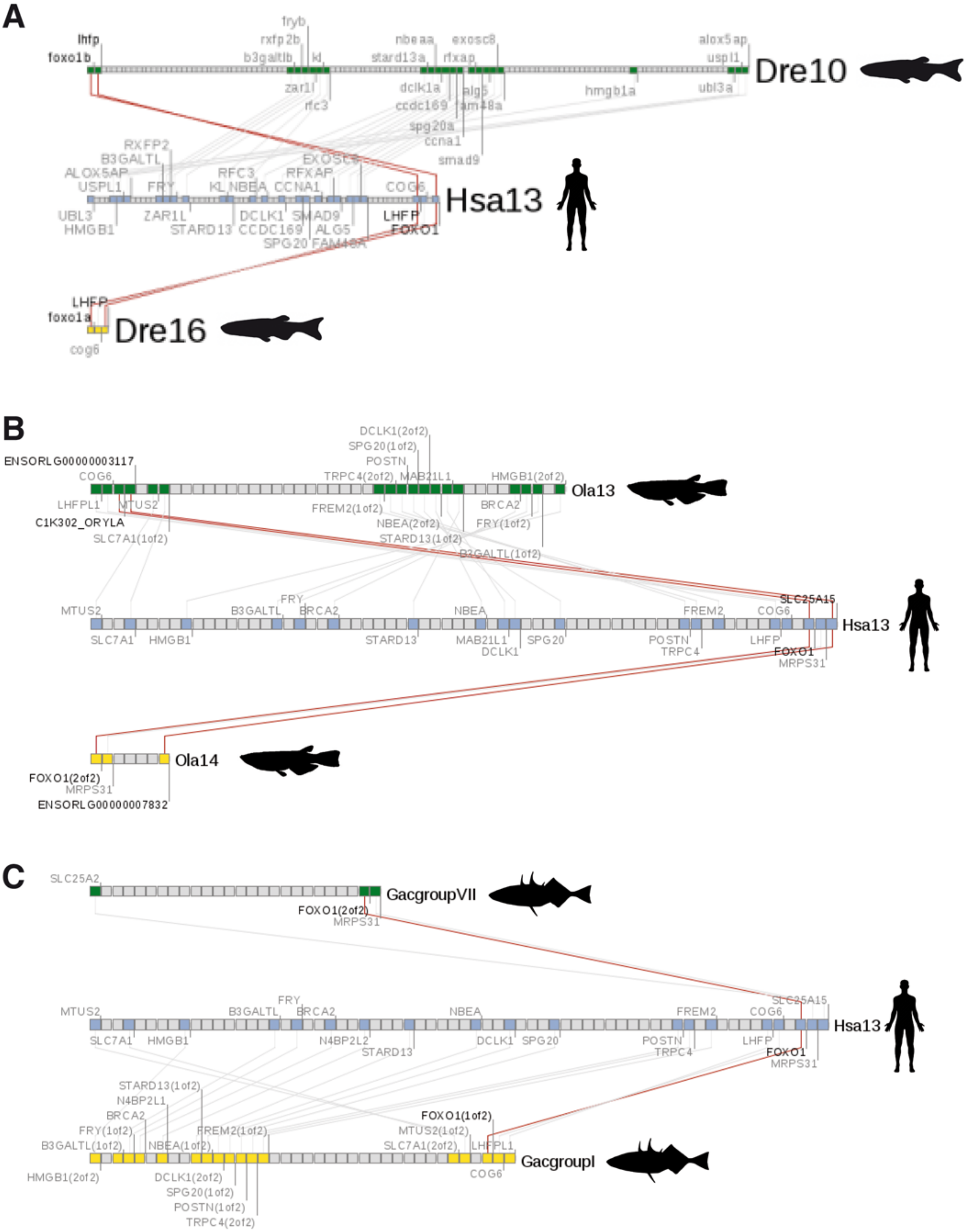

Figure S1: Orthologous pairwise clusters involving the *Foxo1* gene. Syntenic cluster output generated using the Synteny Database using the (A) *Danio rerio* (Zebrafish), (B) *Oryzias latipes* (Medaka) or (C) *Gasterosteus aculeatus* (Stickleback) as source genomes and the Human as outgroup. Sliding window size: 50 genes.

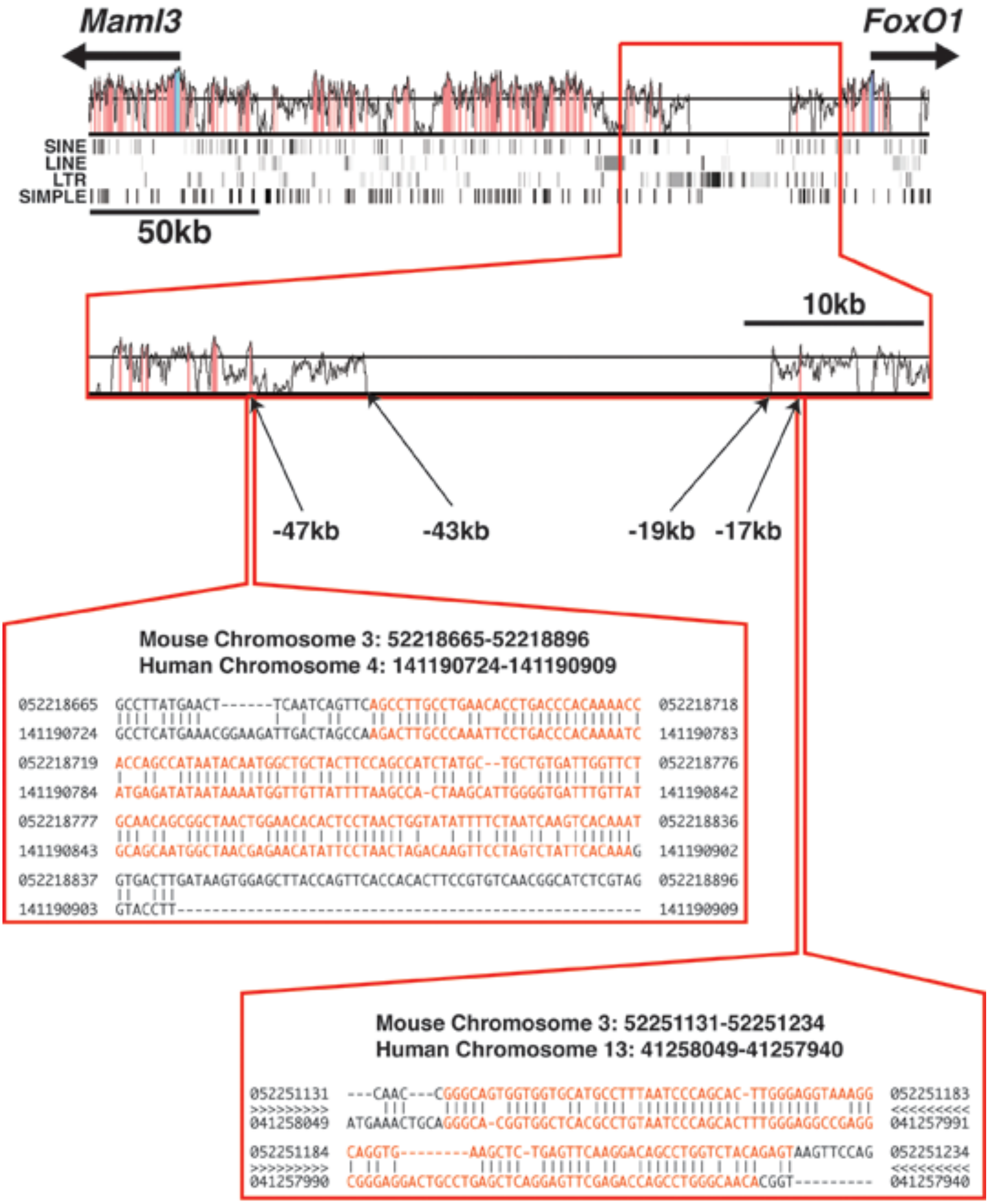

Figure S2: Conservation analysis across the *FoxO1-Maml3* intergenic region. VISTA BROWSER (http://pipeline.lbl.gov; 2017) analysis of the region of interest. The base genome is mouse (mm10), compared to human (hg19). Red peaks are ECRs based on standard parameters (70% identity over 100bp; 100bp sliding window). Underneath, the output from the UCSC Genome Browser showing the location repeats. Note the large LTR in the region without homology. To the left of the LTR, all peaks correspond to sequences in human chr4, while peaks situated to the right have are homologous to human sequences on chr13. The first of such sequences is shown in the alignments underneath, indicating the chromosomal positions; in red, conserved bases. The positions of the furthermost conserved sequences in relation to the *FoxO1* TSS are indicated.

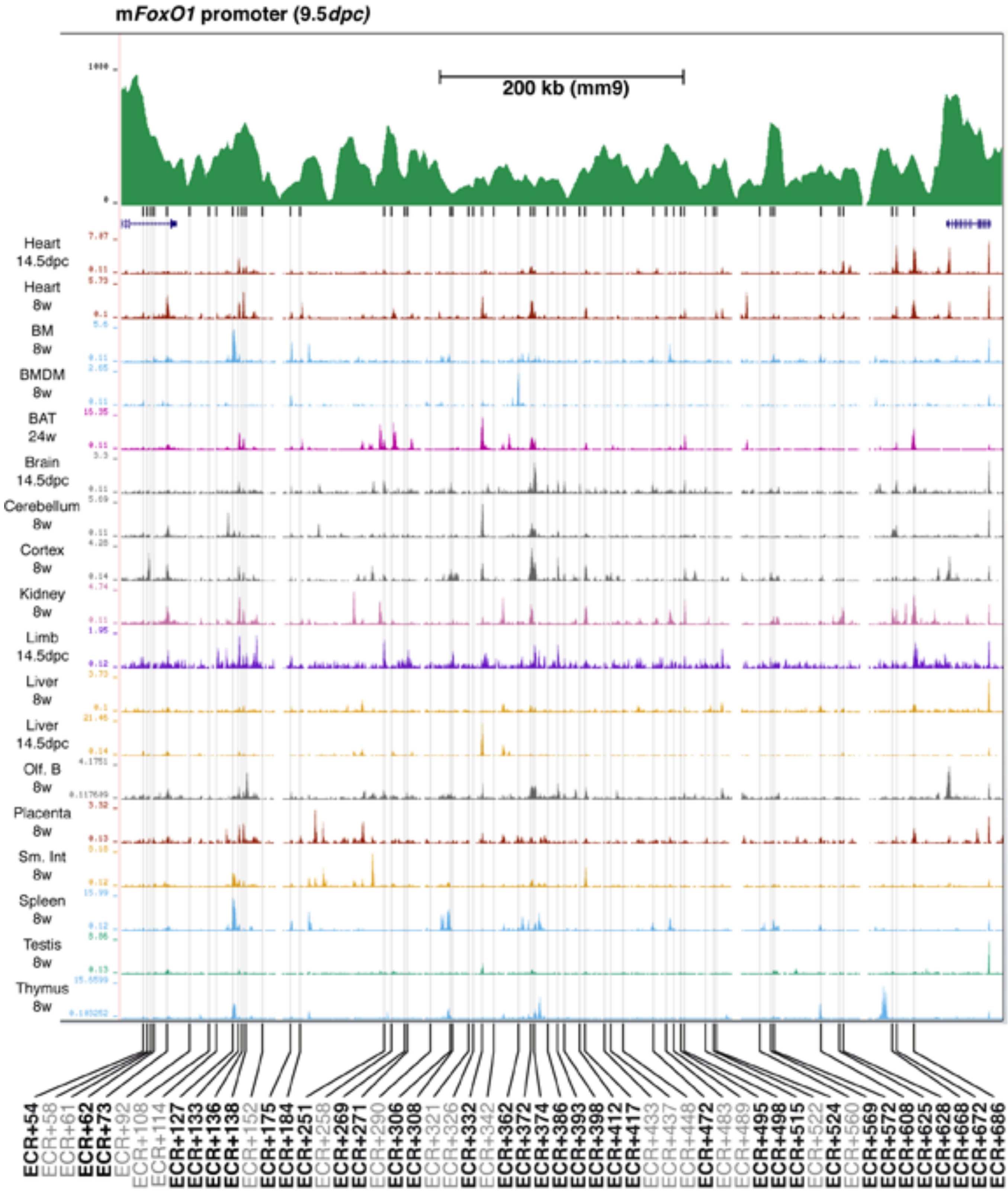

Figure S3: ECRs identified in the *Foxo1* region downstream of the RMS breakpoint and associated H3K27ac marks. In green, the 4C-seq profile when using the *Foxo1* promoter as a viewpoint on chromatin obtained from 9.5 d*pc* mouse embryos. Note how most peaks co-localise with ECRs throughout the landscape. H3K27ac marks in different mouse tissues are also included. Those ECRs co-localising with strong marks are indicated in bold.

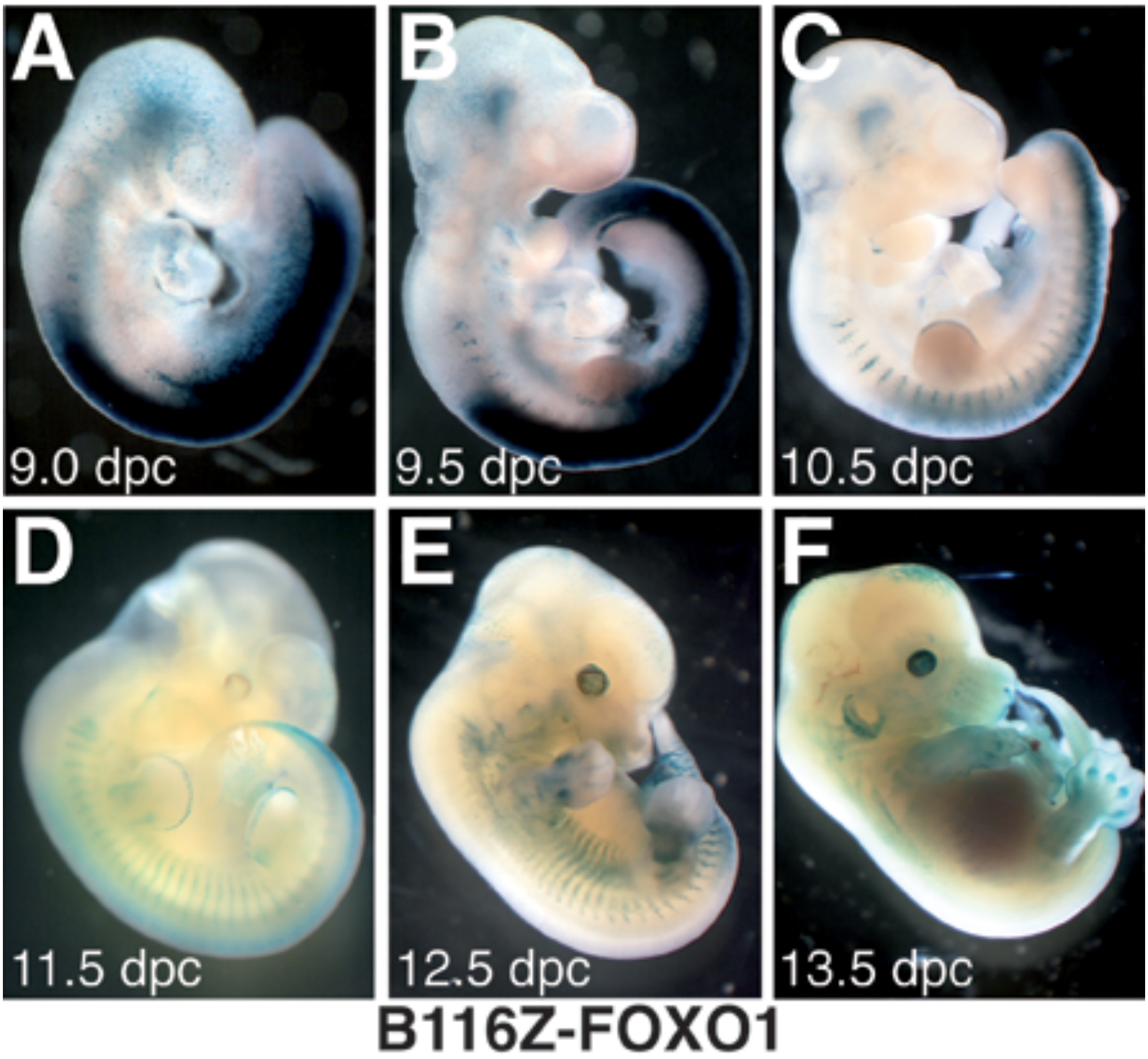

Figure S4: Time-course of embryos carrying the B116Z-Foxo1 reporter construct. Expression starts before 9.0 d*pc* (A) in the neural tube, neural crest and migrating neural crest. At 9.5 d*pc* (B), high levels of transgene expression are detected in the neural tube, the mesonephros, and the vitelin vein, central myotome, head neural crest cells and cells migrating into the forelimb. At 10.5 d*pc* (C), neural tube and mesonephros expression is downregulated, maintained in the foregut and the myotome of cervical and thoracic somites and activated in the AER. At 11.5 d*pc* (D), expression is detected in the myotome, AER, pharyngeal region of the foregut and the posterior half of the neural tube. At 12.5 d*pc* (E), expression is mainly restricted to skeletal musculature, with activation in retina, lens vesicle, pre-cartilage primordia of forelimbs, umbilical cord and neural tube in the tail region. At 13.5 d*pc* (F), the transgene is downregulated in all skeletal muscles, maintained in pre-cartilage primordia of phalangeal bones, and activated in the nasal pits, head epidermis, and follicles of the vibrissae and sinus of sensory facial hairs.

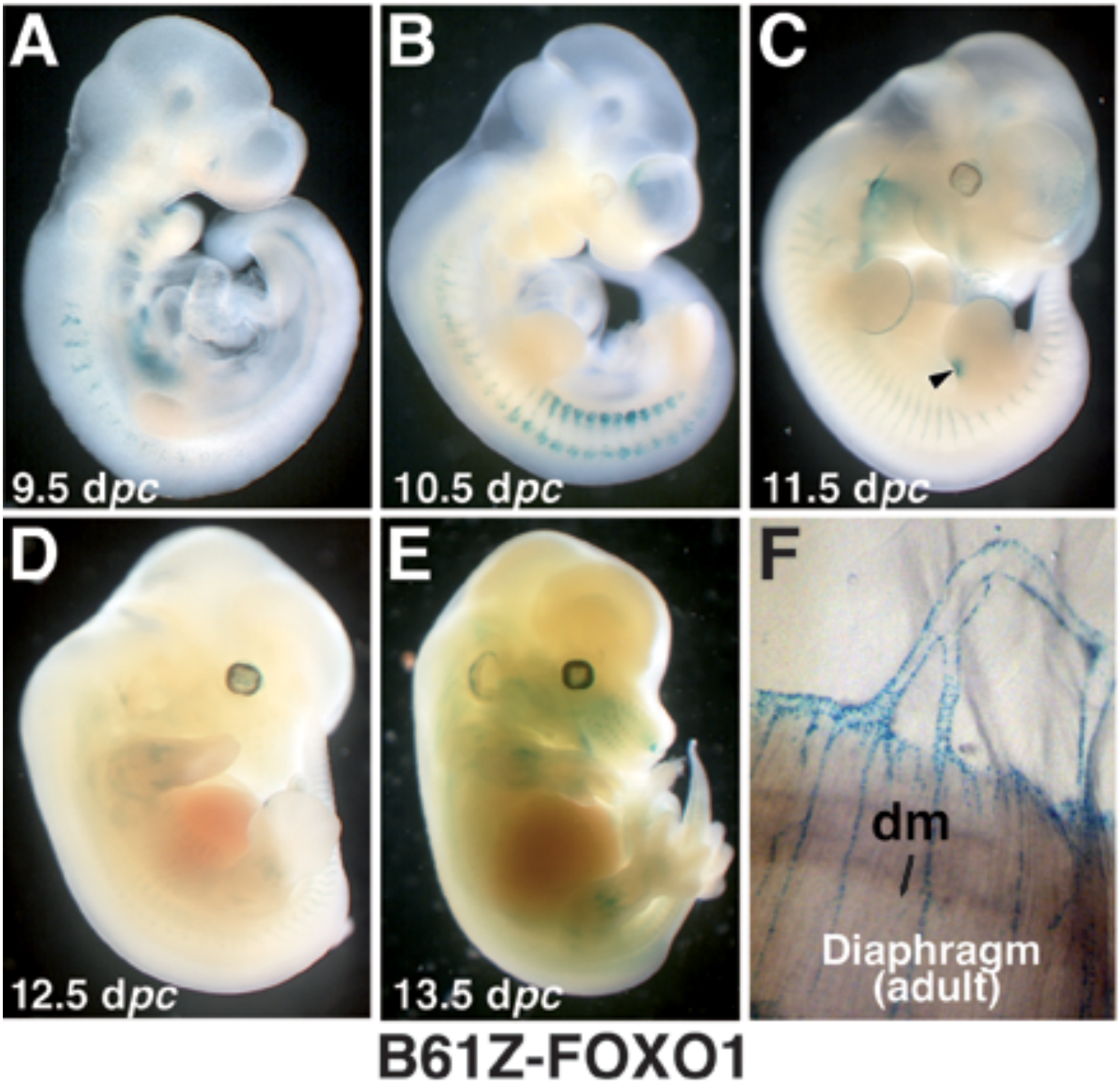

Figure S5: Time-course of embryos carrying the B61Z-Foxo1 reporter construct. Expression is observed at 9.5 d*pc* (A) in the myotome of cervical somites, and fore- and hind-gut, at 10.5 d*pc* (B), in cervical and thoracic somites, gut, vitelin vein and AER, at 11.5 d*pc* (C), in myotome, AER, pharyngeal region, and a hindlimb rostral domain (arrowhead). By 12.5 d*pc* (D), the transgene is downregulated. At 13.5 d*pc* (E), expression corresponds to skeletal muscle, retina, lens vesicle, and nasal pits. In the adult (F), there is strong vasculature expression.

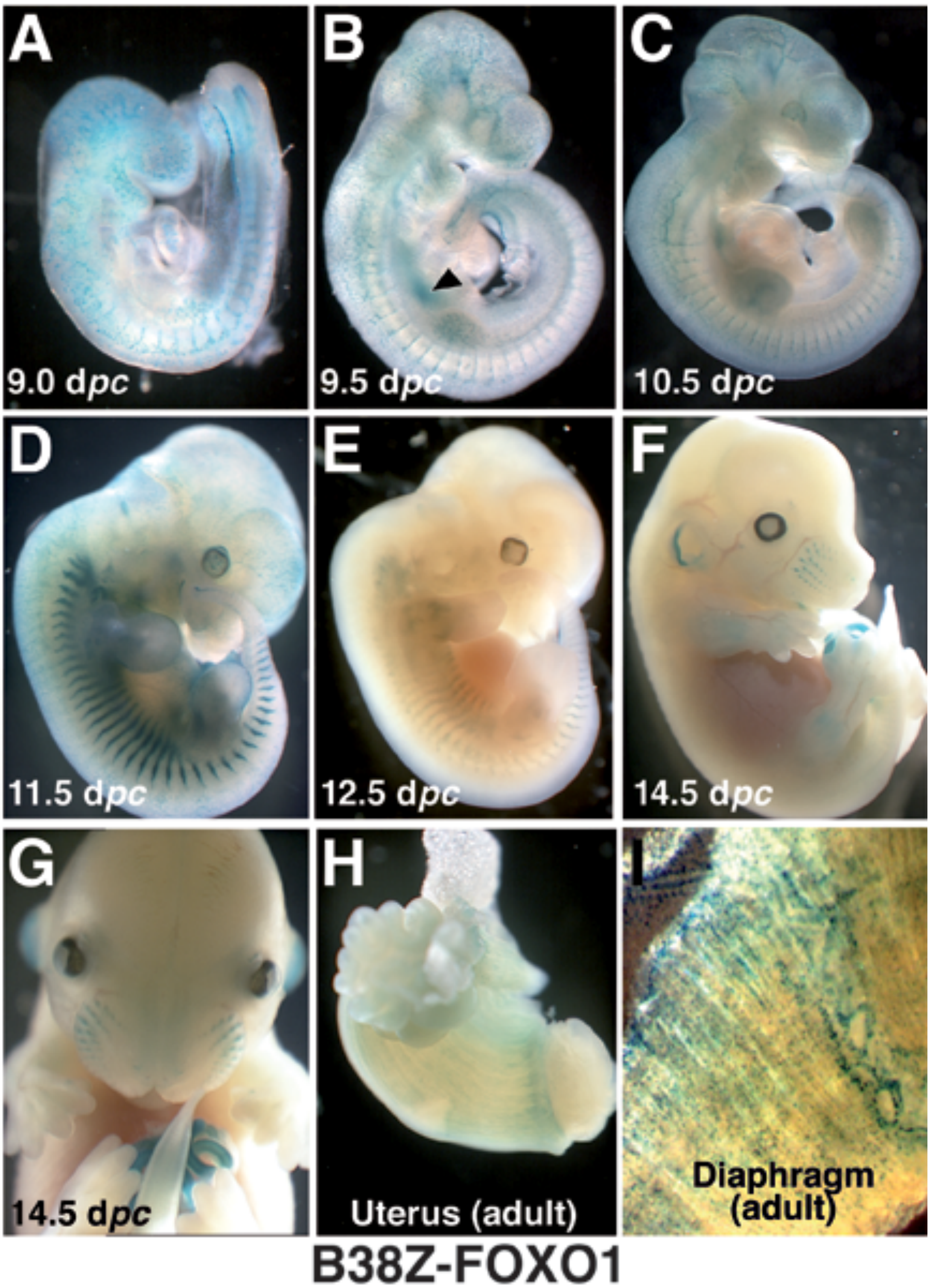

Figure S6: Time-course of embryos carrying the B38Z-Foxo1 reporter construct. Expression is observed at 9.0 d*pc* (A) in vascular precursors, at 9.5 d*pc* (B) in all the vasculature and the foregut (arrowhead). At 10.5 d*pc* (C), expression is maintained in vascular precursors and foregut. At 11.5 d*pc* (D), vascular expression downregulates and myotomal expression is upregulated. At 12.5 d*pc* (E) expression is mainly in skeletal muscle lineage. At 14.5 d*pc* (F), expression is faintly maintained in limb musculature, upregulated in ear cartilage, nasal pits, vibrissae, sensory facial hair follicles, and umbilical cord (G). In the adult, expression is observed in smooth muscle (H), skeletal muscle and vasculature (I). *dm*: diaphragm muscle.

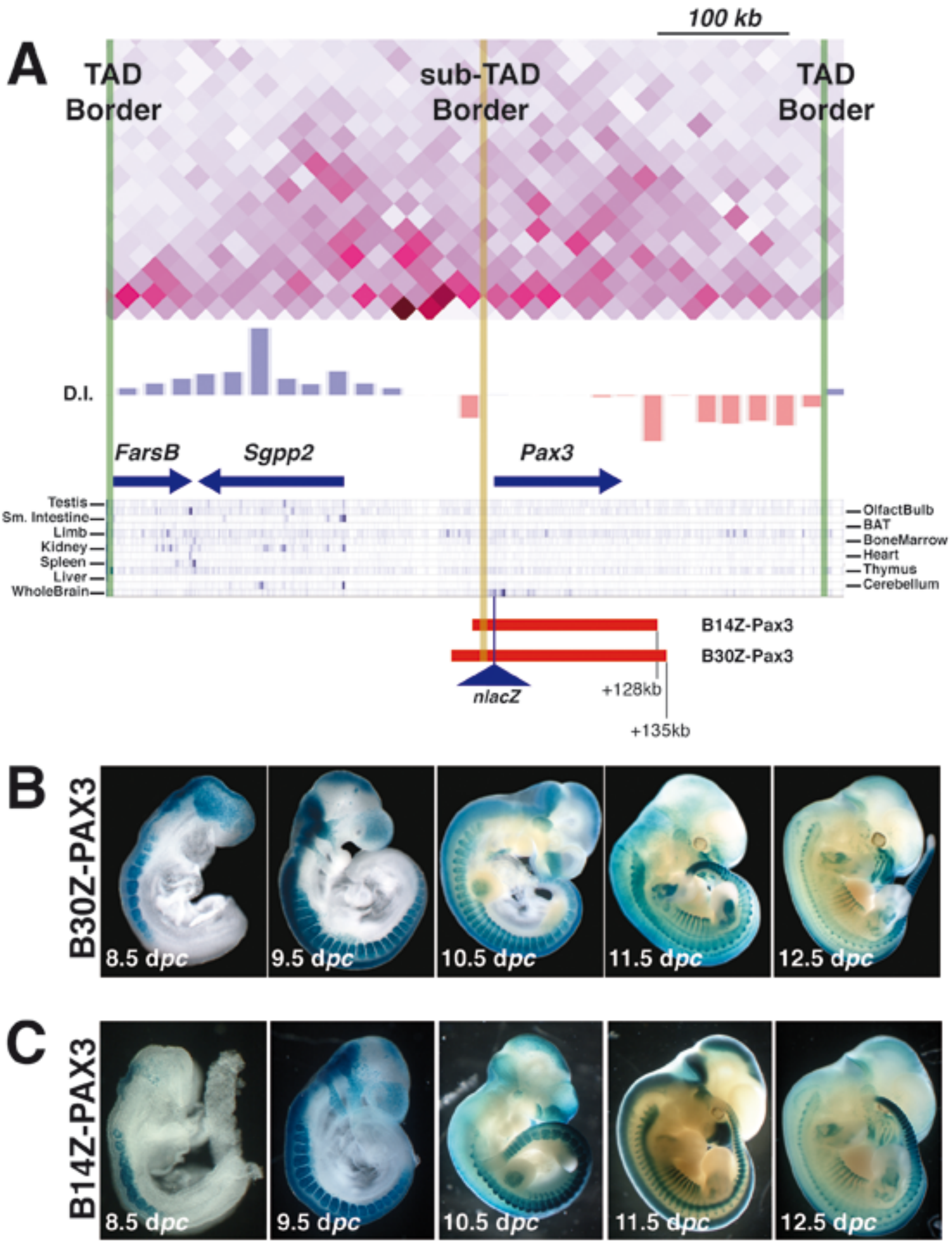

Figure S7. Recapitulation of *Pax3* endogenous expression pattern by a BAC carrying 30kb of upstream sequences. (A) Detail of the Hi-C data from mouse ES cells between the TAD borders (green boxes) and showing the position of the subTAD border and the D.I. analysis output. H3K27ac marks in different mouse tissues are shown underneath, as well as the position of the three coding genes in the region and the relative positions of the two BAC clones used in the study. The 5' ends of the clones cross the subTAD border. (B) Expression patterns of B30Z-PAX3 and (C) B14Z-PAX3 from 8.5 d*pc* to 12.5 d*pc*.

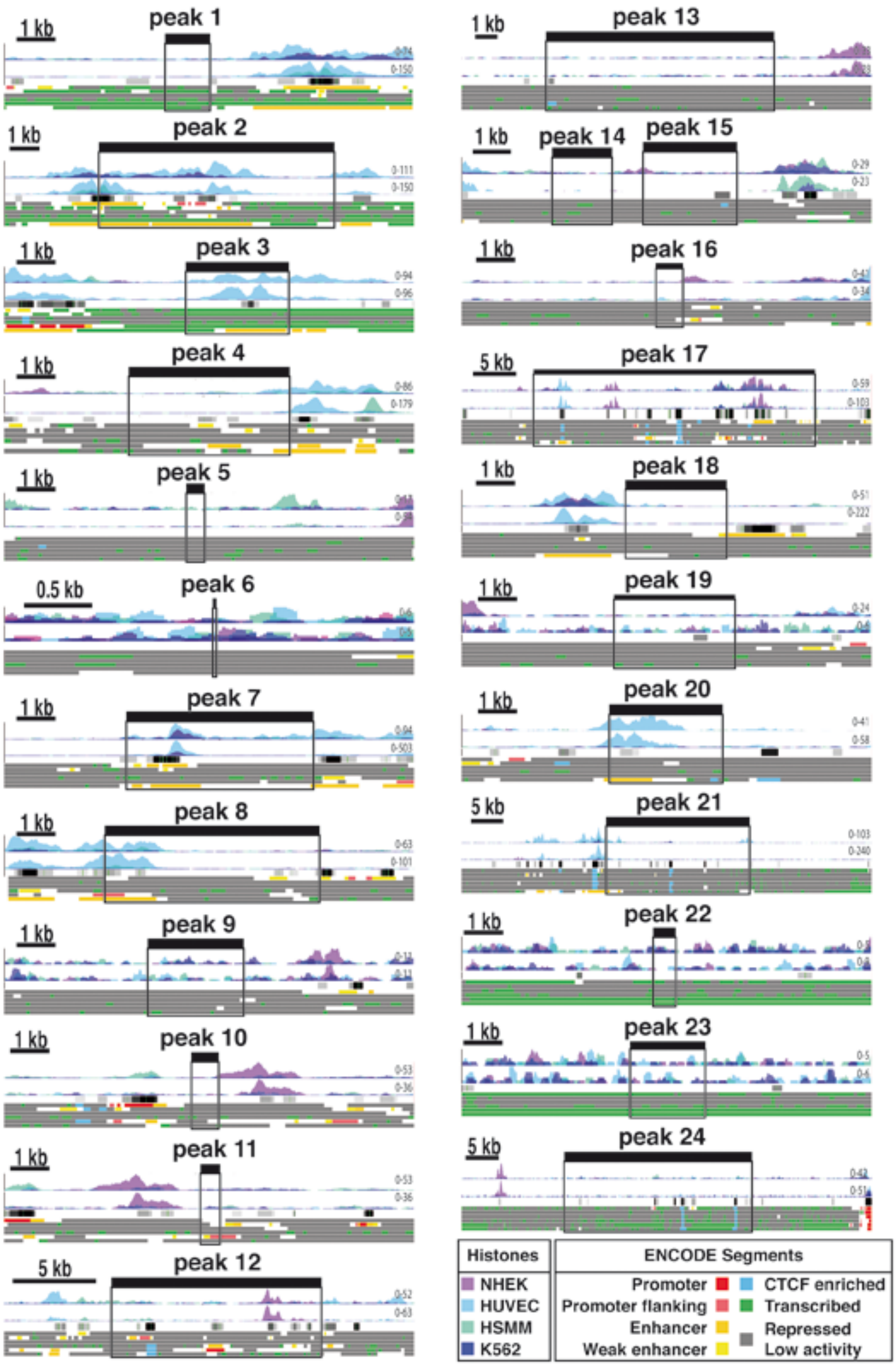

Figure S8. Peaks of interaction established by the *PAX3* promoter at the *FOXO1* locus in RMS cells. Graphical representation of the 4C-seq contacts identified by the Peak Calling algorithm. The peaks are outlined as a box; underneath are representations of H3K4me1, H3K27ac, Transcription Factor ChIP-seq and predicted ENCODE regions. A colour key in included.
