# Supplementary material for: Regulatory landscape fusion in rhabdomyosarcoma through interactions between the PAX3 promoter and FOXO1 regulatory elements
Source: Genome Biol. 2017 Jun 14;18:106. doi: 10.1186/s13059-017-1225-z (PMC5470208; doi:10.1186/s13059-017-1225-z)
Supplement: Supplementary file 2 — Oligonucleotides used in this work. Table S2. Comparison of the TAD borders called at the PAX3 and FOXO1 human loci. Table S3. Interaction peaks between the PAX3 promoter and regions within the FOXO1 locus in RMS cells. Table S4. Number of times the defined TAD boundaries appeared in the iteration. (PDF 296 kb) [file 13059_2017_1225_MOESM2_ESM.pdf]

Table S1 Oligonucleotides used in this work.

Application	Name	Sequence (5' to 3')
**Recombineering**	pFoxHAF+*Apa*I *	tatatagggcccACTCGGAGGCTCCTTAGACACC
	pFoxHAR+*Apa*I *	tatatagggcccAAAGTCGGTGCTGACAGCGGAC
	Linker sequence	CATGCTAGCATATACATATGATATAAGATCTGTAC
	pPax3-5HAF+*Eag*I *	cggCCGGGACGGTCTCCTCCTCG
	pPax3-5HAR+LINK †	gatctaacatatgaagctagCCTGGGGGCAGCTTCGCTCGC
	pPax3-3HAR+*Eag*I *	cggccgATTCAAAAGTGAACGCAGCCCGC
	pPax3-3HAF+LINK †	tagcttcatatgttagatctATGACCACGCTGGCCGGCGC
**Recombineering Rh30 deletion cassette**	LoxP511-Foxo1 ‡	CTGCTGTTTGCCGTGTTACGGGTTCTTCTCGGTTAGCTCCATC ATGCCAGAGGTGTACAGAGGAGGGGAAGGTGataacttcgtataatgta tactatacAGTGGGTGGGAGTCGATTTGTAGAAGGGTAAGACAGC CAGGGCTCAACTCTTTATTGTAATAATTCAAGAAAGC
	Loxp511-Pax3 ‡	GGGAGAGGGGAGAGGGGGAAAGGGGAAGGGAAAAGGGGA TGGAGAGAGAGAGAAAGAGAGAGAGACCCAGtgaagttcctatacttt ctagagaataggaacttcataacttcgtataatgtatgctatacgaagttatCTGAGGACTAC GATGAATGAGTTATAAGAGCACAAATTAAGAGAATTGCTAG TCTTTGGGACTATATAAA
**LD-PCR**	Pax3-LD1	GTATGAACAGATACAATTTCTCCCTCTTTTCATC
	Pax3-LD2	GCCAAATGTAGGAAAATGTTAGTGCTGTATTC
	Pax3-LD3	TGAGAAGAGTTAACAGCAGGTAATGTCATTCC
	Pax3-LD4	TAATAATGGAAAACCCACGTGGAGACCTAAC
	Pax3-LD5	ACACCTGTCCTATTTCATGGATAATCTACTGAGG
	Pax3-LD6	AGTCCCGTGTTTCTAGACAGACGATTTGCTG
	Pax3-LD7	TGGCCTAAAAGAAAACATGATGGTTGACAATC
	Foxo1-LD1	AATACCTACAAAAGAATTTCTGTGCCACTGACTTG
	Foxo1-LD2	CAAAGAATGCAATGGCACAAACTTTAACATTC
	Foxo1-LD3	TCCCTTCTATCTGAAAAATCTTCGAAAATAAAAC
	Foxo1-LD4	AAAACCTGGGAGGAGATCACAGATTCAAAGTC
	Foxo1-LD5	AAAAATTCAGCTGAAGGATCTTTCTCAACAGTAG
	Foxo1-LD6	TATTCAAACTCACGCCTAAAGAAATTCTTCAG
	Foxo1-LD7	TAGGAGCACAGAAAAGTGTAAAATACCTCAAAGG
	Foxo1-LD8	CACTAACATAAGTCAAATAATGAATGCTGCTG
	Foxo1-LD9	AAAGAAGGGGGAAAGAAGACAGGAGAAGAGTG
	Foxo1-LD10	TAAAAGTCACTCATCACAAACTTCCTGTTTAAAG
	Foxo1-LD11	GTACAATACTTCTCTGCTTGCAAAACTTCCTATTC

**4C-seq**	4C-mPax3_RS	TGTTTTCGTCTTACTTGGGGATC
	4C-mPax3_NRS	CTGCCTGCGCTCATTTGT
	4C-hPAX3_RS	AGAAGGGCCGGGCTGATC
	4C-hPax3_NRS	GGCCACTTCCCTATCCGC
	4C-mFoxO1_RS	CTTCACCATCCACTCGTAGATC
	4C-mFoxO1_NRS	CAGCGGTTGCAGAAAGTT
	4C-VP1_RS	TGGTCAAGTTGCTGTCGATC
	4C-VP1_NRS	CTCCTTGATAAACAACAGTAGAGG
	4C-VP2_RS	GGACAGGTAACAGAATCAGATC
	4C-VP2_NRS	AGAAACAAGGATAGTCCAAACC
	4C-VP4_RS	TAGACTGACCACAGGAAGATC
	4C-VP4_NRS	GATGGAAACATCTGAACAAGG
	4C-VP5_RS	GCATTTGCATTCAATGGATC
	4C-VP5_NRS	GGATATTTCTGGGAGTTGAAGC
	4C-VP6_RS	TTCAAACCCATCAGGAGATC
	4C-VP6_NRS	CTACCTAGGTTGGGCTCTGG
	4C-VP7_RS	GGCATGTGGTTCTAGGTGATC
	4C-VP7_NRS	GTTTCCTGCTGTTGGAGTGC
	4C-VP8_RS	CCACACAGTGTTTACTTTGATC
	4C-VP8_NRS	TGTATGAGTTAATGTTATTTAAGAAGG
	4C-VP9_RS	GGTCATCAGGAGTTATGTGTGATC
	4C-VP9_NRS	TCCTTCATGGTCTGGACTGC

(*) small caps indicate the engineered restriction sites and additional flanking sequences

(†) small caps indicate engineered linker sequence

(‡) small caps indicate the engineered LoxP511 sites

Table S2 Comparison of the TAD borders called at the *PAX3* and *FOXO1* human loci.

Cell type / Tissue *	TAD border upstream *hPAX3*	Distance between *hPAX3* borders (kb) †	TAD border downstream *hFOXO1*	Distance between *hFOXO1* borders (kb) †
RMS	chr2:223491756-223531756		chr13:40242000-40282000	
**Fully differentiated cells**				
GM12878	chr2:223520000-223560000	**27.4**	chr13:40200000-40240000	**29.9**
IMR90	chr2:223440000-223480000	52.6	chr13:40280000-40320000	50.1
**ESC/ESC derived cell lines**				
H1	chr2:223520000-223560000	**27.4**	chr13:40280000-40320000	50.1
MES	chr2:223520000-223560000	**27.4**	chr13:40280000-40320000	50.1
MSC	chr2:223520000-223560000	**27.4**	chr13:40240000-40280000	**10.1**
NPC	chr2:223560000-223600000	67.4	chr13:40240000-40280000	**10.1**
TRO	chr2:223520000-223560000	**27.4**	chr13:40200000-40240000	**29.9**
**Adult tissues**				
AD	chr2:223520000-223560000	**27.4**	chr13:40280000-40320000	50.1
AO	chr2:223440000-223480000	52.6	chr13:40200000-40240000	**29.9**
BL	chr2:223480000-223520000	**12.6**	chr13:40160000-40200000	69.9
CO	chr2:223440000-223480000	52.6	chr13:40240000-40280000	**10.1**
HC	chr2:223480000-223520000	**12.6**	chr13:40240000-40280000	**10.1**
LI	chr2:223480000-223520000	**12.6**	chr13:40200000-40240000	**29.9**
LG	chr2:223480000-223520000	**12.6**	chr13:40240000-40280000	**10.1**
LV	chr2:223480000-223520000	**12.6**	chr13:40240000-40280000	**10.1**
OV	chr2:223480000-223520000	**12.6**	chr13:40160000-40200000	69.9
PA	chr2:223440000-223480000	52.6	chr13:40200000-40240000	**29.9**
PO	chr2:223440000-223480000	52.6	chr13:40240000-40280000	**10.1**
RV	chr2:223480000-223520000	**12.6**	chr13:40240000-40280000	**10.1**
SB	chr2:223960000-224000000	467.4	chr13:40280000-40320000	50.1
SX	chr2:223920000-223960000	427.4	chr13:40240000-40280000	**10.1**

This table compares the borders identified in this study with respect to those defined in a panel of 21 different cell types and tissues (30). Overlapping borders (within the +/-20 kb resolution range) are highlighted in bold lettering.

(*) Cell type / Tissue: RMS, rhabdomyosarcoma cell line; GM12878, lymphoblastoid cell line; IMR90, fetal lung fibroblast cell line; H1, embryonic stem cell line; MES, ESC derived mesendoderm cell line; MSC, ESC derived mesenchymal stem cells; NPC, neural progenitor cells; TRO, trophoblast-like cells; AD, adrenal gland; AO, aorta; BL, bladder; CO, prefrontal cortex; HC, hippocampus; LI, liver; LG, lung; LV, left ventricle; OV, ovary; PA, pancreas; PO, psoas muscle; RV, right ventricle; SB, small bowel; SX, spleen.

(†) Distance between the centers of the ranges that define these TAD borders in human RMS (this paper) and the cell types and tissues analysed by Schmitt and colleagues (30).

Table S3 Interaction peaks between the *PAX3* promoter and regions within the *FOXO1* locus in RMS cells.

	**Peak no.**	**1**	**2**	**3**	**4**	**5**	**6**	**7**	**8**	**9**	**10**	**11**	**12**	**13**	**14**	**15**	**16**	**17**	**18**	**19**	**20**	**21**	**22**	**23**	**24**
	**Size (bp)**	1139	7993	2648	4128	490	27	4813	5512	2472	681	501	12550	9916	1621	2518	699	32514	2599	3118	2910	22260	586	1946	28962
	**ECRs[a]**		+54, +58, +61		+133			+175, +184					+271					+483, +489, +495, +498							
	**No. of TF binding sites[b]**	1	40	3	5	0	0	55	6	0	0	0	23	1	0	1	0	56	0	2	3	10	0	0	14
	CTCF	y	y										y					y				y			y
	Rad21							y					y	y		y		y				y			y
	**No. of tissues with DNAseI HS[c]**	1	86	16	9	1		79	50	5		4	20	1	1	2		119	1	6	24	63		1	75
	**Tissues with H3K27ac marks[d]**																								
**Expression**	**Tissue**																								
medium	brain	y	y	y	y			y	y									y			y	y		y	y
medium	adipose nuclei	y	y	y	y			y	y				y					y				y			
medium	ovary		y		y			y	y				y	y				y			y	y			y
medium	smooth muscle[e]	y	y		y			y	y				y					y				y			y
medium	lung		y	y	y			y	y				y					y				y			y
medium	skeletal muscle[e]		y	y	y			y	y				y					y							y
medium	HUVEC		y	y				y	y									y			y				
medium	esofhagus		y					y	y									y				y			
medium	spleen		y		y			y	y									y							
medium	aorta				y																				
low	heart[e]		y		y			y	y	y		y	y	y				y				y			y
low	immune system[e]	y	y	y	y			y	y	y								y				y	y		y
low	MSCs		y					y	y				y					y				y			
low	liver		y	y				y	y				y					y				y			
low	sigmoid colon	y	y					y	y									y				y			
low	pancreas		y		y			y	y				y					y							
low	small instestine		y	y				y	y													y			y
low	NHEK		y					y					y					y							
low	HepG2		y		y								y					y							
low	HMEC		y					y					y					y							
low	thymus		y						y																y
low	NHLF								y																y
low	stomach																					y			
no	osteoblast							y	y									y				y			y
N/D	digestive mucosa[e]		y	y				y	y			y						y				y			
N/D	NHDF								y									y							y

[a] ECRs contained within each peak (position relative to *Foxo1* TSS); [b] Experimentally validated TF binding sites derived from ChIP-seq data from the ENCODE project that map within each peak; [c] DNAseI HS master list from ENCODE (125 cell types). In case there are multiple sites within the same peak, only the one present in the highest number of tissues is listed; [d] H3K27ac marks from the ChIP-seq collection at the WashU Epigenome Browser; [e] Smooth muscle: stomach, duodenum, colon and rectal smooth muscle; skeletal muscle: HSMM-derived myoblasts and myotubes, as well as adult skeletal muscle; heart: right and left atrium, right ventricle; immune system: B-, T-cells, natural killer cells, monocytes, hematopoietic stem cells and the lymphoblastoid cell line GM12878; digestive mucosa: rectal and colonic mucosa; y, yes; N/D, not determined; HUVEC, Human Umbilical Vein Endothelial Cells; MSCs, bone-marrow derived Mesenchymal Stem Cells; NHEK, Normal Human Epidermal Keratinocytes; HMEC, Human Mammary Epithelial Cells; NHLF, Normal Human Lung Fibroblasts; NHDF, Normal Human Dermal Fibroblasts.

Table S4 Number of times the defined TAD boundaries appeared in the iteration.

PAX3 Human: TAD size from 15 to 40

Boundary	Times appeared	Comment
Chr2: 222,451,756	25 (100%)	TAD boundary
Chr2: 222,871,756	25 (100%)	TAD boundary
Chr2: 223,171,756	25 (100%)	Sub-TAD boundary
Chr2: 223,511,756	25 (100%)	TAD boundary
Chr2: 223,871,756	25 (100%)	TAD boundary

FOXO1 human: TAD size from 20 to 50

Boundary	Times appeared	Comment
Chr13: 39,502,000	30 (100%)	TAD boundary
Chr13: 40,262,000	30 (100%)	TAD boundary
Chr13: 41,362,000	30 (100%)	TAD boundary
Chr13: 41,902,000	30 (100%)	TAD boundary

Pax3 Mouse: TAD size from 15 to 30

Boundary	Times appeared	Comment
Chr1: 77,530,000	15 (100%)	TAD boundary
Chr1: 77,950,000	15 (100%)	TAD boundary
Chr1: 78,200,000	13 (86.7%)	Sub-TAD boundary
Chr1: 78,490,000	15 (100%)	TAD boundary
Chr1: 78,740,000	13 (86.7%)	TAD boundary

Foxo1 mouse: TAD size from 20 to 40

Boundary	Times appeared	Comment
Chr3: 51,220,000	20 (100%)	TAD boundary
Chr3: 51,500,000	20 (100%)	TAD boundary
Chr3: 52,060,000	20 (100%)	TAD boundary
Chr3: 52,780,000	20 (100%)	TAD boundary

RMS Translocation: TAD size from 30 to 90

Boundary	Times appeared	Comment
Chr2: 223,815,327	60 (100%)	TAD boundary
Chr13: 40,249,897	58 (96%)	TAD boundary
Chr2: 223,512,553	45 (75%)	TAD boundary
Chr13: 39,799,255	31 (52%)	TAD boundary

Column 1 shows the predicted chromosomal location of the boundaries identified by D.I. analysis (±20 kb); Column 2 shows the number of times it was selected as a boundary in the iterative process and the percentage it represents; Column 3 shows the interpretation of the defined boundary.
